# Iron-catalyzed decarboxylative alkenylation of cycloalkanes with arylvinyl carboxylic acids via a radical process

**DOI:** 10.3762/bjoc.9.197

**Published:** 2013-08-21

**Authors:** Jiancan Zhao, Hong Fang, Jianlin Han, Yi Pan

**Affiliations:** 1 School of Chemistry and Chemical Engineering, State of Key Laboratory of Coordination, Nanjing University, Nanjing, 210093, China; 2 Institute for Chemistry & BioMedical Sciences, Nanjing University, Nanjing, 210093, China

**Keywords:** alkenylation, cycloalkanes, decarboxylative, Fe(acac)_3_, free radical, sp^3^ C–H bonds

## Abstract

A Fe(acac)_3_-catalyzed decarboxylative coupling of 2-(aryl)vinyl carboxylic acids with cycloalkanes was developed by using DTBP as an oxidant through a radical process. This reaction tolerates a wide range of substrates, and products are obtained in good to excellent yields (71–95%). The reaction also shows excellent stereoselectivity, and only *trans*-isomers are obtained.

## Introduction

Direct C–H functionalization has become one of the most useful and attractive tools in organic chemistry because it can construct carbon–carbon or carbon–heteroatom bonds in a highly atom economical manner [1–8]. Among all these C–H functionalization methods, the direct C(sp^3^)–H functionalization attracts particular attention due to its low reactivity and challenging activation [9–11]. In previous studies, numerous transition-metal-catalyzed processes, such as Pd [12–18], Cu [19–23], Ru [24–27], Rh [28–31], Co [32–34], Au [35–36], Ir [37–39], Fe [40–43] and other metals [44–47], have been developed for sp^3^ C–H activation reactions. Additionally, metal-free methodologies, which use TBHP, PhI(OAc)_2_, TBAI, I_2_ or Lewis/Brønsted acids, have also been employed for cross-dehydrogenative coupling reactions [48–57].

Owing to the general low reactivity of cycloalkane C(sp^3^)–H bonds, the direct alkenylation of cycloalkanes with high selectivity and stereospecificity remains a great challenge and attracted a lot of attention in the past years. In 1996, the Fuchs group described the alkenylation of cyclohexane by a radical reaction with vinyl triflone [58]. In 2003, the Yao group reported that styryl cycloalkanes were prepared based on a radical substitution of cyclohydrocarbon units to (*E*)-β-nitrostyrenes by using the radical initiator benzoyl peroxide [59]. Recently, the Liu group developed a copper-catalyzed decarboxylative coupling of vinylic carboxylic acids with simple alcohols and ethers in high yields. Cycloalkanes were also investigated in this catalytic system, though only moderate yields were obtained [60].

Several other groups have also found that Pd, Ag or Cu could catalyze the decarboxylative coupling of various aromatic, alkenyl, and alkynyl carboxylic acids [61–68]. Our group was surprised to find that low-cost Fe(acac)_3_ could catalyze the direct alkenylation of cyclohexane sp^3^ C–H bonds by decarboxylative couplings with high efficiency.

## Results and Discussion

We initiated our investigation by reacting cinnamic acid (**1a**, 0.3 mmol) with cyclohexane (**2a**, 2 mL) in the presence of iron(II) chloride tetrahydrate (20 mol %) and 2.0 equiv of di-*tert*-butyl peroxide (DTBP) as the oxidant at 120 °C under nitrogen, which provided the expected (*E*)-(2-cyclohexylvinyl)benzene (**3a**), but in a moderate 54% yield (Table 1, entry 1). The use of aqueous TBHP as oxidant instead of DTBP reduced the yield to only 38% (Table 1, entry 2). With the help of 1,10-phenanthroline (30 mol %) as the ligand, the yield could be slightly improved to 68% (Table 1, entry 3). Iron(III) acetylacetonate provided a superior yield (91%) compared to the other Fe salts such as FeCl_3_, ferrocene, Fe_2_O_3_ and Fe_3_O_4_ tested (Table 1, entries 4–8). Application of other oxidants such as K_2_S_2_O_8_, H_2_O_2_ (30% aqueous solution) or TBPB did not afford any improvements (Table 1, entries 9–11). A decreased loading of Fe(acac)_3_ to 10 mol % or an increased amount of DTBP to 5.0 equiv and a lower temperature (110 °C), decreased the yield to 63%, 69% and 79%, respectively (Table 1, entries 12–14). The reaction did not proceed without the iron catalyst or DTBP (Table 1, entries 15 and 16).

**Table 1:** Optimization of typical reaction conditions.^a^

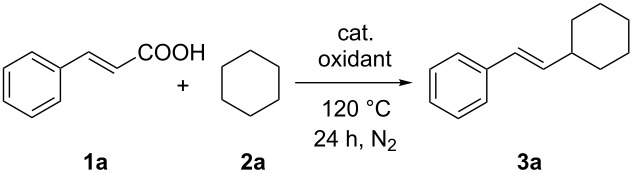			

Entry	cat. (mol %)	oxidant	yield (%)^b^

1	FeCl_2_·4H_2_O (20)	DTBP	54
2	FeCl_2_·4H_2_O (20)	TBHP	38
3	FeCl_2_·4H_2_O (20)	DTBP	68^c^
4	FeCl_3_ (20)	DTBP	Trace
5	Ferrocene (20)	DTBP	74
6	Fe_2_O_3_ (20)	DTBP	78
7	Fe_3_O_4_ (20)	DTBP	80
8	Fe(acac)_3_ (20)	DTBP	91
9	Fe(acac)_3_ (20)	K_2_S_2_O_8_	N.D.
10	Fe(acac)_3_ (20)	H_2_O_2_^d^	21
11	Fe(acac)_3_ (20)	TBPB	49
12	Fe(acac)_3_ (10)	DTBP^e^	63
13^f^	Fe(acac)_3_ (20)	DTBP	69
14	Fe(acac)_3_ (20)^g^	DTBP	79
15	Fe(acac)_3_ (20)	–	N.D.
16	–	DTBP	N.D.

^a^Catalytic conditions: cinnamic acid (0.3 mmol), cyclohexane (2 mL), iron catalyst (20 mol %), oxidant (2.0 equiv), 120 °C, 24 h, N_2_. ^b^Isolated yields based on cinnamic acid. ^c^Using 1,10-phenanthroline (30 mol %) as the ligand. ^d^30% aqueous solution. ^e^5 equiv. ^f^12 h. ^g^110 °C.

**Scheme 1:**
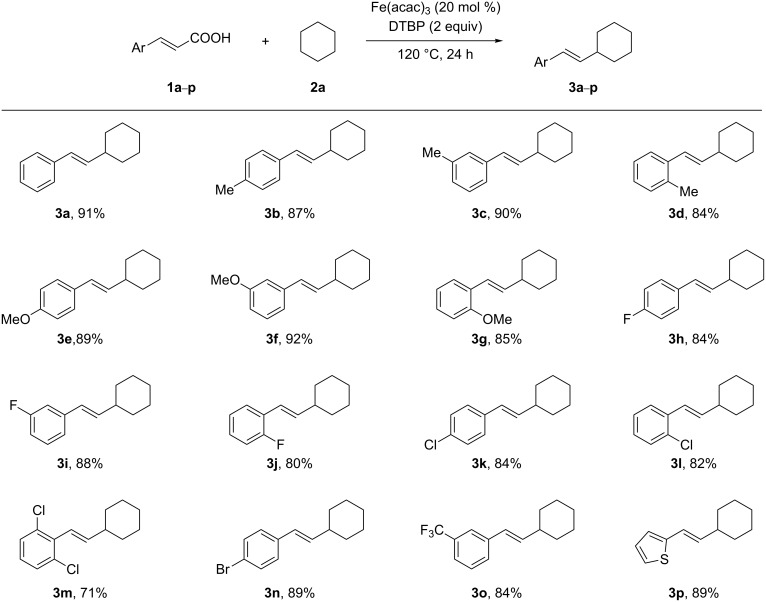
Fe(acac)_3_-catalyzed alkenylation of cyclohexane. Catalytic conditions: cinnamic acid (**1**) (0.3 mmol), cyclohexane (2 mL), Fe(acac)_3_ (20 mol %), DTBP (2.0 equiv), 120 °C, 24 h, N_2_. Yields are isolated yields based on cinnamic acid.

We then examined the substrate scope and limitation of the procedure by reacting cyclohexane with a variety of substituted cinnamic acid derivatives under the optimized conditions (Table 1, entry 8). As shown in Scheme 1, almost all of the tested substrates worked well in this reaction. Several substituents on the aromatic ring were tolerated and the position of these substituents showed almost no effect on the chemical yield. We also observed that electron-donating substituents, such as methyl or methoxy groups at any position of the ring, efficiently took part in the reaction with a slightly decreased yield in case of ortho-substituted products (Scheme 1, **3b**–**g**). Furthermore, a reaction of 2,6-disubstituted cinnamic acid **1m** lead to the expected product **3m**, which was obtained in a lower yield due to steric hindrance. In addition, heteroaryl-substituted acrylic acids can also be efficiently converted under these conditions. This was shown by the reaction of 3-(thiophen-2-yl)acrylic acid (**1p**) with cyclohexane furnishing the product **3p** in 89% yield. In general, the stereoselectivity of this reaction was excellent and only *trans*-isomers were obtained in all cases.

**Scheme 2:**
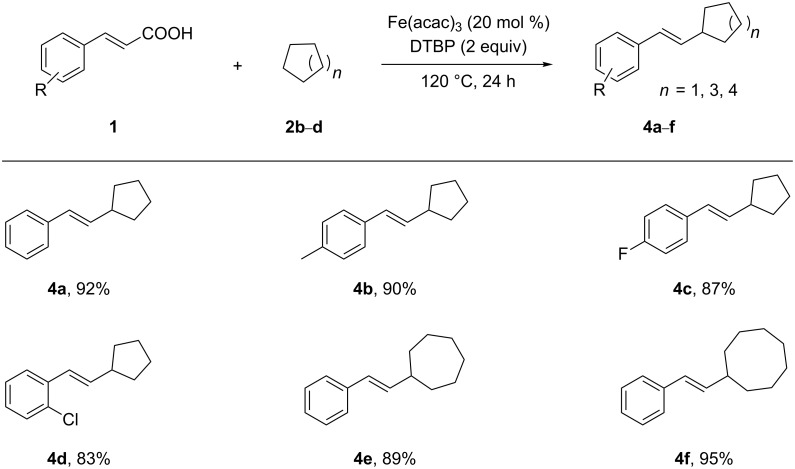
Fe(acac)_3_-catalyzed alkenylation of cyclopentan, cycloheptane and cyclooctane. Catalytic conditions: cinnamic acid (**1**) (0.3 mmol), cycloalkanes (2.0 mL), Fe(acac)_3_ (20 mol %), DTBP (2 equiv), 120 °C, 24 h, N_2_. Yields are isolated yields based on cinnamic acid.

Next, other cycloalkanes, including cyclopentane, cycloheptane and cyclooctane, were reacted with different cinnamic acids **1**, giving products **4a**–**f** in 83–95% chemical yield (Scheme 2). As already shown for cyclohexane as a substrate, the reaction of other cycloalkanes performs equally well with a variety of cinammic acid derivatives under these conditions. It is noteworthy, that the decarboxylative cross-coupling with cyclooctane showed a higher efficiency than with smaller cycloalkanes.

**Scheme 3:**
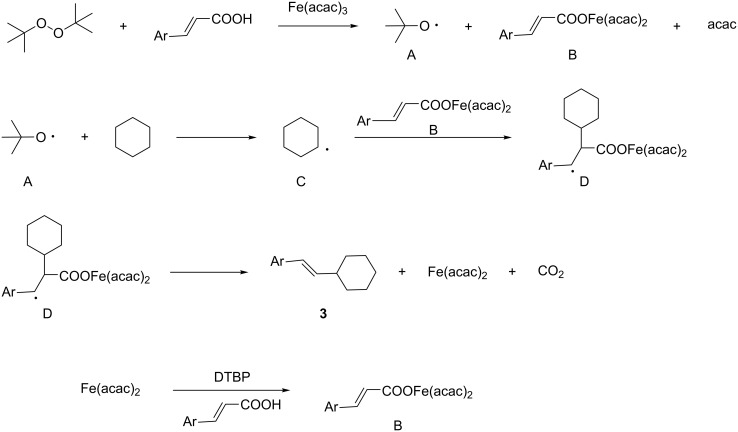
A plausible pathway for the reaction.

Finally two control experiments were carried out to shed light on the reaction mechanism. Addition of the radical scavenger 2,2,6,6-tetramethylpiperidine *N*-oxide (TEMPO) or azobisisobutyronitrile (AIBN) completely inhibited the reaction, and almost no desired product was obtained. Based on these results and literature reports [69–70], a plausible mechanism for the radical oxidative coupling is illustrated in Scheme 3. At the beginning, Fe-catalyzed cleavage of DTBP by Fe(III) in the presence of cinnamic acid, gives *tert*-butoxy radical **A**, intermediate **B** and one acac. Next, a cyclohexane radical **C** is generated by the reaction between *tert*-butoxy radical **A** and cyclohexane. Subsequently, addition of cyclohexane radical **C** to the α-position of the double bond in **B** gives intermediate **D**. Finally, the radical intermediate **D** is oxidatively decarboxylated by Fe(III) to give product **3**, Fe(II) and carbon dioxide. The Fe(III) catalyst is then reformed via DTBP oxidation [71].

## Conclusion

In conclusion, an efficient procedure for the Fe(acac)_3_-catalyzed direct alkenylation of sp^3^ C–H bonds of cycloalkanes with DTBP as an oxidant has been reported. This method provides a useful strategy for the stereospecific synthesis of substituted *E*-alkenes. Various cinnamic acids and cycloalkanes are well-tolerated in this catalytic system with good to excellent chemical yields. The mechanism of this reaction has also been studied, and a radical mechanism was proposed. Further studies on the alkenylation of other sp^3^ C–H substrates are currently investigated in our laboratory.

## Experimental

**General procedure for the iron-catalyzed decarboxylative alkenylation of cycloalkanes:** To a Schlenk tube equipped with a magnetic stir bar were added Fe(acac)_3_ (21.2 mg, 0.06 mmol) and cinnamic acid (0.3 mmol) under a nitrogen atmosphere. Cycloalkane (2.0 mL, 15–25 mmol) and DTBP (di-*tert*-butyl peroxide, 0.6 mmol, 113 μL) were added under a nitrogen atmosphere and the resulting reaction mixture was stirred at 120 °C for 24 h. After cooling to room temperature and removal of volatiles, the products were isolated by flash column chromatography (PE).

## Supporting Information

File 1 Experimental details and spectral data.
