# Supplementary material for: Iron-catalyzed decarboxylative alkenylation of cycloalkanes with arylvinyl carboxylic acids via a radical process
Source: Beilstein J Org Chem. 2013 Aug 21;9:1718–23. doi: 10.3762/bjoc.9.197 (PMC3778410; doi:10.3762/bjoc.9.197)
Supplement: File 1 — Experimental details and spectral data. [file Beilstein_J_Org_Chem-09-1718-s001.pdf]

Supporting Information
for
Iron-catalyzed decarboxylative alkenylation of cycloalkanes with arylvinylic carboxylic acids via a radical process

Jiancan Zhao[1], Hong Fang[1], Jianlin Han*[1,2] and Yi Pan*[1]

Address: [1]School of Chemistry and Chemical Engineering, State of Key Laboratory of Coordination, Nanjing University, Nanjing, 210093, China and [2]Institute for Chemistry & BioMedical Sciences, Nanjing University, Nanjing, 210093, China

Email: Jianlin Han* - hanjl@nju.edu.cn; Yi Pan* - yipan@nju.edu.cn

*Corresponding author

Experimental details and spectral data

Index

1. Experimental Section S2
   General Information S2
   General Procedure S2
2. Characterization data for compounds **3a–p** and **4a–f** S3
3. ^{1}H and ^{13}C NMR spectra **3a–p** and **4a–f** S9
4. References S31

1. Experimental Section

General Information: All reactions were carried out under a nitrogen atmosphere. Column chromatography was carried out on silica gel (300–400 mesh). Analytical thin–layer chromatography (TLC) was performed on glass plates of Silica Gel GF-254 with detection by UV light. Standard techniques for synthesis under inert gas atmosphere by using gasbag and Schlenk glassware equipped with an 8 mm PTFE vacuum stop-cock (Synthware) were employed. All starting materials and reagents were commercially available. ^{1}H and ^{13}C NMR spectra were recorded on a Bruker AVANCE400M spectrometer. The chemical shift references were as follows: (^{1}H) $CDCl_3$, 7.26 ppm ($CHCl_3$); (^{13}C) $CDCl_3$, 77.00 ppm ($CDCl_3$). HRMS spectra were carried out at Micromass GCT (TOF MS EI^{+}). Melting points were determined on a Melt-Temp apparatus (X-4) from Beijing Fukai Electro-optic Instrument Plant and are uncorrected.

General Procedure: To a Schlenk tube equipped with a magnetic stir bar were added $Fe(acac)_3$ (21.2 mg, 0.06 mmol) and cinnamic acid (0.3 mmol) under a nitrogen atmosphere. Cycloalkane (2.0 mL, 15–25 mmol) and DTBP (di-*tert*-butyl peroxide, 0.6 mmol, 113 μL) were added under nitrogen and the resulting reaction mixture was kept stirring at 120 °C for 24 h. After cooling to room temperature and removal of volatiles the products were isolated by flash column chromatography (PE).

Products **3a**, **3e**, **3h**, **3j**, **3k**, **3p**, **4a**, **4c**, **4e**, **4f**, **3b**, **3l**, **3d**, **3n** and **4b**, are known compounds. ^{1}H and ^{13}C NMR spectra of these compounds have been confirmed to be identical to those of known samples (**3a**, **3e**, **3h**, **3j**, **3k**, **3p**, **4a**, **4c**, **4e** and **4f**) [1], (**3b** and **3l**) [2], **3d** [3], **3n** [4], **4b** [5]).

2. Characterization data for 3a–p and 4a–f.

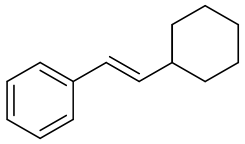

(*E*)-(2-Cyclohexylvinyl)benzene(3a, known compound): Colorless oil, ^{1}H NMR (400 MHz, $CDCl_3$) δ 7.33–7.22 (m, 4H), 7.16-7.12 (m, 1H), 6.32 (d, *J* = 16.1 Hz, 1H), 6.15 (dd, *J* = 16.0, 6.9 Hz, 1H), 2.18–2.00 (m, 1H), 1.86–1.59 (m, 5H), 1.40–1.07 (m, 5H). ^{13}C NMR (100 MHz, $CDCl_3$) δ 138.02, 136.70, 128.38, 127.26, 126.66, 125.91, 41.12, 32.93, 26.16, 26.03.

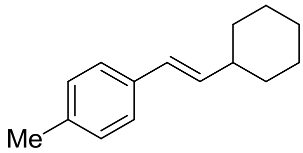

(*E*)-1-(2-Cyclohexylvinyl)-4-methylbenzene(3b, known compound): Colorless oil, ^{1}H NMR (400 MHz, $CDCl_3$) δ 7.22 (d, *J* = 8.1 Hz, 2H), 7.06 (d, *J* = 8.0 Hz, 2H), 6.29 (d, *J* = 16.0 Hz, 1H), 6.10 (dd, *J* = 16.0, 6.9 Hz, 1H), 2.29 (s, 3H), 2.13-2.05 (m, 1H), 1.84–1.63 (m, 5H), 1.35–1.12 (m, 5H). ^{13}C NMR (100 MHz, $CDCl_3$) δ 136.29, 135.73, 135.26, 129.09, 127.06, 125.81, 41.11, 33.01, 26.19, 26.06, 21.06.

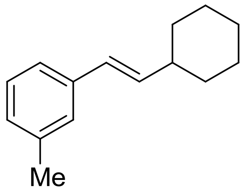

(*E*)-1-(2-Cyclohexylvinyl)-3-methylbenzene(3c): Colorless oil, ^{1}H NMR (400 MHz, $CDCl_3$) δ 7.20–7.06 (m, 3H), 6.97–6.95 (m, 1H), 6.29 (d, *J* = 16.0 Hz, 1H), 6.13 (dd, *J* = 16.0, 6.9 Hz, 1H), 2.29 (s, 3H), 2.18–2.03 (m, 1H), 1.86–1.60 (m, 5H), 1.40–1.08 (m, 5H). ^{13}C NMR (100 MHz, $CDCl_3$) δ 137.96, 137.79, 136.47, 128.29, 127.46, 127.33, 126.61, 123.11, 41.14, 32.97, 26.17, 26.04, 21.33. HRMS (TOF MS EI^+) *m/z* calcd for [$C_{15}H_{20}$] 200.1565, found 200.1553.

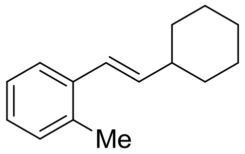

(*E*)-1-(2-Cyclohexylvinyl)-2-methylbenzene(3d, known compound): Colorless oil, ^{1}H NMR (400 MHz, $CDCl_3$) δ 7.63 (d, *J* = 7.0 Hz, 1H), 7.39–7.29 (m, 3H), 6.77 (d, *J* = 15.8 Hz, 1H), 6.26 (dd, *J* = 15.8, 7.0 Hz, 1H), 2.54 (s, 3H), 2.44–2.30 (m, 1H), 2.11–1.85 (m, 5H), 1.65–1.34 (m, 5H). ^{13}C NMR (100 MHz, $CDCl_3$) δ 138.10, 137.10, 134.81, 130.05, 126.63, 125.92, 125.34, 125.09,

41.43, 33.08, 26.18, 26.05, 19.74.

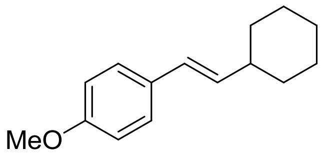

(*E*)-1-(2-Cyclohexylvinyl)-4-methoxybenzene(3e, known compound): Colorless oil, ^{1}H NMR (400 MHz, $CDCl_3$) δ 7.17 (d, *J* = 8.7 Hz, 1H), 6.72 (d, *J* = 8.7 Hz, 1H), 6.19 (d, *J* = 16.0 Hz, 1H), 5.94 (dd, *J* = 16.0, 6.9 Hz, 1H), 3.67 (s, 3H), 2.04-1.96 (m, 1H), 1.76–1.53 (m, 5H), 1.25–1.04 (m, 5H). ^{13}C NMR (100 MHz, $CDCl_3$) δ 158.57, 134.65, 130.84, 126.94, 126.55, 113.83, 55.15, 41.07, 33.06, 26.17, 26.06.

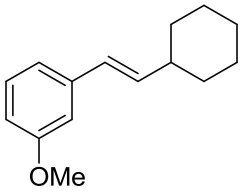

(*E*)-1-(2-Cyclohexylvinyl)-3-methoxybenzene(3f): Colorless oil,^{1}H NMR (400 MHz, $CDCl_3$) δ 7.18 (t, *J* = 7.9 Hz, 1H), 6.96–6.86 (m, 2H), 6.72 (dd, *J* = 8.1, 2.1 Hz, 1H), 6.30 (d, *J* = 16.0 Hz, 1H), 6.16 (dd, *J* = 16.0, 6.8 Hz, 1H), 3.78 (s, 3H), 2.12-2.06 (m, 1H), 1.85–1.63 (m, 5H), 1.34–1.14 (m, 5H). ^{13}C NMR (100 MHz, $CDCl_3$) δ 159.76, 139.51, 137.07, 129.33, 127.13, 118.60, 112.35, 111.22, 55.07, 41.08, 32.89, 26.14, 26.00. HRMS (TOF MS EI^+) *m/z* calcd for [$C_{15}H_{20}O$] 216.1514, found 216.1510.

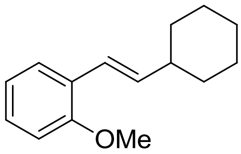

(*E*)-1-(2-Cyclohexylvinyl)-2-methoxybenzene(3g): Colorless oil, ^{1}H NMR (400 MHz, $CDCl_3$) δ 7.41 (dd, *J* = 7.6, 1.1 Hz, 1H), 7.14 (ddd, *J* = 8.8, 7.7, 1.1 Hz, 1H), 6.87 (t, *J* = 7.4 Hz, 1H), 6.80 (d, *J* = 8.2 Hz, 1H), 6.69 (d, *J* = 16.1 Hz, 1H), 6.14 (dd, *J* = 16.1, 7.0 Hz, 1H), 3.78 (s, 3H), 2.23–2.05 (m, 1H), 1.88–1.61 (m, 5H), 1.38–1.11 (m, 5H). ^{13}C NMR (100 MHz, $CDCl_3$) δ 156.27, 137.27, 127.66, 127.04, 126.12, 121.72, 120.54, 110.70, 55.31, 41.53, 33.04, 26.17, 26.05. HRMS (TOF MS EI^+) *m/z* calcd for [$C_{15}H_{20}O$] 216.1514, found 216.1505.

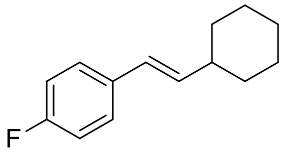

(*E*)-1-(2-Cyclohexylvinyl)-4-fluorobenzene(3h, known compound): White solid, mp 34–35 °C, ^{1}H NMR (400 MHz, $CDCl_3$) δ 7.50 (d, *J* = 8.2 Hz, 2H), 7.38 (d, *J* = 8.2 Hz, 2H), 6.34 (d, *J* = 16.1 Hz, 1H), 6.25 (dd, *J* = 16.0, 6.6 Hz, 1H), 2.17-2.09 (m, 1H), 1.87–1.64 (m, 5H), 1.33-1.16 (m, 5H). ^{13}C NMR (100 MHz, $CDCl_3$) δ 141.64 (d, *J* = 1.2 Hz), 139.56, 128.61 (d, *J* = 32.3 Hz), 126.18, 126.05, 125.36 (q, *J* = 3.9 Hz), 41.23, 32.76, 26.12, 25.99.

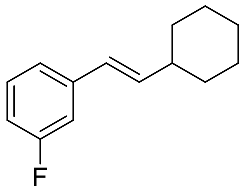

(*E*)-1-(2-Cyclohexylvinyl)-3-fluorobenzene(3i): Colorless oil, ^{1}H NMR (400 MHz, $CDCl_3$) δ 7.20 (td, *J* = 7.9, 6.1 Hz, 1H), 7.04 (ddd, *J* = 10.4, 8.1, 4.8 Hz, 2H), 6.88–6.82 (m, 1H), 6.29 (d, *J* = 16.0 Hz, 1H), 6.16 (dd, *J* = 15.9, 6.8 Hz, 1H), 2.14-2.06 (m, 1H), 1.87–1.62 (m, 5H), 1.35–1.11 (m, 5H). ^{13}C NMR (100 MHz, $CDCl_3$) δ 163.15 (d, *J* = 244.7 Hz), 140.50 (d, *J* = 7.7 Hz), 138.17, 129.77 (d, *J* = 8.5 Hz), 126.31 (d, *J* = 2.5 Hz), 121.82 (d, *J* = 2.6 Hz), 113.42 (d, *J* = 21.4 Hz), 112.31 (d, *J* = 21.7 Hz), 41.08, 32.81, 26.12, 25.99. HRMS (TOF MS EI$^+$) *m/z* calcd for [$C_{14}H_{17}F$] 204.1314, found 204.1320.

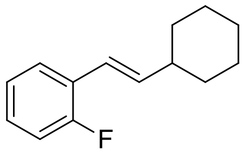

(*E*)-1-(2-Cyclohexylvinyl)-2-fluorobenzene(3j, known compound): Colorless oil, ^{1}H NMR (400 MHz, $CDCl_3$) δ 7.42-7.38 (m, 1H), 7.16–6.93 (m, 3H), 6.50 (d, *J* = 16.2 Hz, 1H), 6.22 (dd, *J* = 16.1, 7.0 Hz, 1H), 2.16-2.09 (m, 1H), 1.87–1.61 (m, 5H), 1.39–1.10 (m, 5H). ^{13}C NMR (100 MHz, $CDCl_3$) δ 160.00 (d, *J* = 248.0 Hz), 139.26 (d, *J* = 4.1 Hz), 127.83 (d, *J* = 8.3 Hz), 126.85 (d, *J* = 4.0 Hz), 125.76 (d, *J* = 12.3 Hz), 123.88 (d, *J* = 3.5 Hz), 119.61 (d, *J* = 3.9 Hz), 115.50 (d, *J* = 22.3 Hz), 41.52, 32.84, 26.14, 26.00.

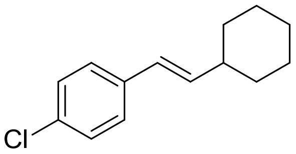

(*E*)-1-Chloro-4-(2-cyclohexylvinyl)benzene(3k, known compound): Colorless oil, ^{1}H NMR (400 MHz, $CDCl_3$) δ 7.25-7.17 (m, 4H), 6.27 (d, *J* = 16.0, 1H), 6.13 (dd, *J* = 16.0, 6.8 Hz, 1H), 2.14-2.06 (m, 1H), 1.80-1.65 (m, 5H), 1.32-1.14 (m, 5H). ^{13}C NMR (100 MHz, $CDCl_3$) δ 137.46, 136.54, 132.20, 128.50, 127.11, 126.09, 41.10, 32.84, 26.12, 25.99.

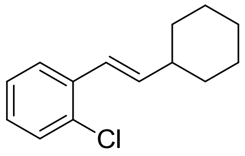

(*E*)-1-Chloro-2-(2-cyclohexylvinyl)benzene(3l, known compound): Colorless oil, ^{1}H NMR (400 MHz, $CDCl_3$) δ 7.47 (dd, *J* = 7.7, 1.4 Hz, 1H), 7.29 (dd, *J* = 7.9, 1.1 Hz, 1H), 7.17–7.04 (m, 2H), 6.72 (d, *J* = 15.9 Hz, 1H), 6.13 (dd, *J* = 15.9, 7.0 Hz, 1H), 2.18-2.13 (m, 1H), 1.87–1.61 (m, 5H), 1.38–1.10 (m, 5H). ^{13}C NMR (100 MHz, $CDCl_3$) δ 139.56, 136.05, 132.66, 129.51, 127.68, 126.62, 126.46, 123.62, 41.31, 32.83, 26.13, 25.97.

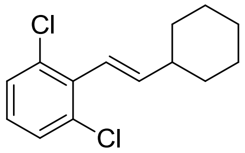

(*E*)-1,3-Dichloro-2-(2-cyclohexylvinyl)benzene(3m): Colorless oil, ^{1}H NMR (400 MHz, $CDCl_3$) δ 7.25 (d, *J* = 8.0 Hz, 2H), 7.00 (t, *J* = 8.0 Hz, 1H), 6.28 (d, *J* = 16.4 Hz, 1H), 6.14 (dd, *J* = 16.3, 6.7 Hz, 1H), 2.24-2.16 (m, 1H), 1.89–1.64 (m, 5H), 1.40–1.17 (m, 5H). ^{13}C NMR (100 MHz, $CDCl_3$) δ 145.16, 135.49, 134.34, 128.23, 127.38, 120.98, 41.52, 32.51, 26.17, 25.89. HRMS (TOF MS EI^{+}) *m/z* calcd for [$C_{14}H_{16}Cl_2$] 254.0629, found 254.0625.

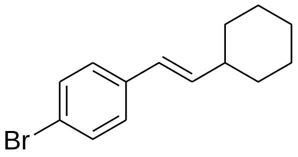

(*E*)-1-Bromo-4-(2-cyclohexylvinyl)benzene (3n, known compound): White solid, mp 39–40 °C, ^{1}H NMR (400 MHz, $CDCl_3$) δ 7.36 (d, *J* = 8.5 Hz, 2H), 7.16 (d, *J* = 8.4 Hz, 2H), 6.25 (d, *J* = 16.1 Hz, 1H), 6.13 (dd, *J* = 16.0, 6.7 Hz, 1H), 2.13-2.05 (m, 1H), 1.87–1.54 (m, 5H), 1.38–1.04 (m, 5H).^{13}C NMR (100 MHz, $CDCl_3$) δ 137.54, 136.95, 131.41, 127.44, 126.14, 120.28, 41.08, 32.79, 26.11, 25.98.

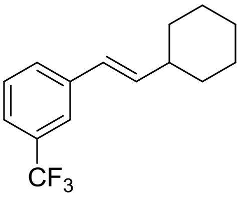

(*E*)-1-(2-Cyclohexylvinyl)-3-(trifluoromethyl)benzene(3o): Colorless oil, ^{1}H NMR (400 MHz, $CDCl_3$) δ 7.57 (s, 1H), 7.46–7.30 (m, 3H), 6.34 (d, *J* = 16.1 Hz, 1H), 6.22 (dd, *J* = 16.0, 6.7 Hz, 1H), 2.18–2.06 (m, 1H), 1.83–1.63 (m, 5H), 1.35–1.11 (m, 5H). ^{13}C NMR (100 MHz, $CDCl_3$) δ 138.85 (d, *J* = 13.6 Hz), 130.90 (q, *J* = 32.0 Hz), 129.13 (d, *J* = 1.1 Hz), 128.81, 126.12, 125.67, 123.22 (q, *J* = 3.8 Hz), 122.96, 122.57 (q, *J* = 3.8 Hz), 41.19, 32.81, 26.13, 26.00. HRMS (TOF MS EI^{+}) *m/z* calcd for [$C_{15}H_{17}F_3$] 254.1282, found 254.1291.

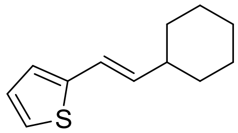

(*E*)-2-(2-Cyclohexylvinyl)thiophene(3p, known compound): Colorless oil, ^{1}H NMR (400 MHz, $CDCl_3$) δ 7.04 (d, *J* = 5.1 Hz, 1H), 6.90 (dd, *J* = 5.1, 3.5 Hz, 1H), 6.84 (d, *J* = 3.6 Hz, 1H), 6.45 (d, *J* = 15.8 Hz, 1H), 6.01 (dd, *J* = 15.8, 6.9 Hz, 1H), 2.12–1.99 (m, 1H), 1.82–1.72 (m, 5H), 1.33–1.11 (m, 5H). ^{13}C NMR (100 MHz, $CDCl_3$) δ 143.38, 136.69, 127.10, 124.13, 122.89, 120.61, 40.89, 32.77, 26.11, 25.97.

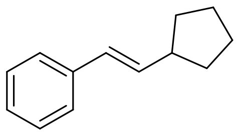

(*E*)-(2-Cyclopentylvinyl)benzene(4a, known compound): Colorless oil, ^{1}H NMR (400 MHz, $CDCl_3$) δ 7.34–7.29 (m, 2H), 7.25 (dd, *J* = 10.4, 4.9 Hz, 2H), 7.14 (ddd, *J* = 8.5, 2.5, 1.2 Hz, 1H), 6.35 (d, *J* = 15.9 Hz, 1H), 6.18 (dd, *J* = 15.8, 7.7 Hz, 1H), 2.62–2.51 (m, 1H), 1.90–1.78 (m, 2H), 1.74–1.52 (m, 4H), 1.44–1.32 (m, 2H). ^{13}C NMR (100 MHz, $CDCl_3$) δ 137.90, 135.56, 128.39, 127.88, 126.65, 125.88, 43.78, 33.18, 25.21.

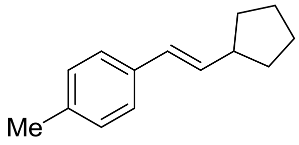

(*E*)-1-(2-Cyclopentylvinyl)-4-methylbenzene(4b, known compound): Colorless oil, ^{1}H NMR (400 MHz, $CDCl_3$) δ 7.48 (d, *J* = 6.5 Hz, 2H), 7.32 (d, *J* = 7.5 Hz, 2H), 6.59 (d, *J* = 15.8 Hz, 1H), 6.39 (ddd, *J* = 15.8, 7.7, 1.7 Hz, 1H), 2.93–2.73 (m, 1H), 2.55 (s, 3H), 2.21–1.58 (m, 8H). ^{13}C NMR (100 MHz, $CDCl_3$) δ 136.15, 135.12, 134.37, 129.05, 127.76, 125.78, 43.78, 33.20, 25.19, 21.01.

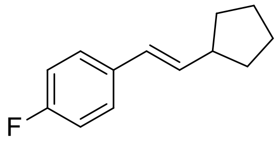

(*E*)-1-(2-Cyclopentylvinyl)-4-fluorobenzene(4c): Colorless oil, ^{1}H NMR (400 MHz, $CDCl_3$) δ 7.31–7.23 (m, 2H), 6.98-6.93 (m, 2H), 6.31 (d, *J* = 15.8 Hz, 1H), 6.09 (dd, *J* = 15.8, 7.8 Hz, 1H), 2.71–2.42 (m, 1H), 1.95–1.22 (m, 48H). ^{13}C NMR (100 MHz, $CDCl_3$) δ 161.82 (d, *J* = 245.5 Hz), 135.35 (d, *J* = 2.2 Hz), 134.07 (d, *J* = 3.2 Hz), 127.27 (d, *J* = 7.8 Hz), 126.71, 115.23 (d, *J* = 21.4 Hz), 43.75, 33.19, 25.20. HRMS (TOF MS EI^+) *m/z* calcd for [$C_{13}H_{15}F$] 190.1158, found 190.1151.

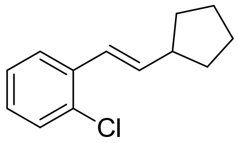

(*E*)-1-Chloro-2-(2-cyclopentylvinyl)benzene(4d): Colorless oil, ^{1}H NMR (400 MHz, $CDCl_3$) δ 7.31 (s, 1H), 7.24–7.06 (m, 3H), 6.27 (d, *J* = 15.8 Hz, 1H), 6.18 (dd, *J* = 15.8, 7.4 Hz, 1H), 2.68–2.41 (m, 1H), 1.92–1.30 (m, 8H). ^{13}C NMR (100 MHz, $CDCl_3$) δ 139.82, 137.17, 134.35, 129.56, 126.63, 126.55, 125.77, 124.14, 43.71, 33.09, 25.20. HRMS (TOF MS EI^+) *m/z* calcd for [$C_{13}H_{15}Cl$] 206.0862, found 206.0861.

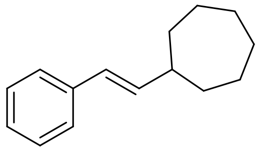

(*E*)-Styrylcycloheptane(4e, known compound): Colorless oil, ^{1}H NMR (400 MHz, $CDCl_3$) δ 7.31 (dd, *J* = 8.3, 1.1 Hz, 2H), 7.25 (dd, *J* = 10.4, 4.9 Hz, 2H), 7.17–7.10 (m, 1H), 6.30 (d, *J* = 16.0 Hz, 1H), 6.19 (dd, *J* = 15.9, 7.4 Hz, 1H), 2.37–2.24 (m, 1H), 1.83–1.78 (m, 2H), 1.68-1.37(m, 10H). ^{13}C NMR (100 MHz, $CDCl_3$) δ 138.09, 137.53, 128.38, 126.70, 126.61, 125.90, 43.20, 34.71, 28.38, 26.25.

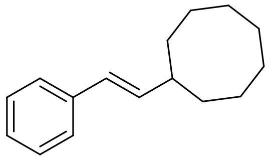

(*E*)-Styrylcyclooctane(4f, known compound): Colorless oil, ^{1}H NMR (400 MHz, $CDCl_3$) δ 7.33–7.28 (m, 2H), 7.24 (dd, *J* = 10.4, 4.9 Hz, 2H), 7.13 (dd, *J* = 10.2, 4.3 Hz, 1H), 6.30 (d, *J* = 16.0 Hz, 1H), 6.18 (dd, *J* = 15.9, 7.4 Hz, 1H), 2.43–2.30 (m, 1H), 1.79–1.44 (m, 14H). ^{13}C NMR (100 MHz, $CDCl_3$) δ 138.08, 137.61, 128.35, 126.88, 126.58, 125.89, 41.29, 31.83, 27.43, 25.98, 25.06.

3. ^{1}H and ^{13}C NMR spectra

^{1}H- and ^{13}C NMR of 3a

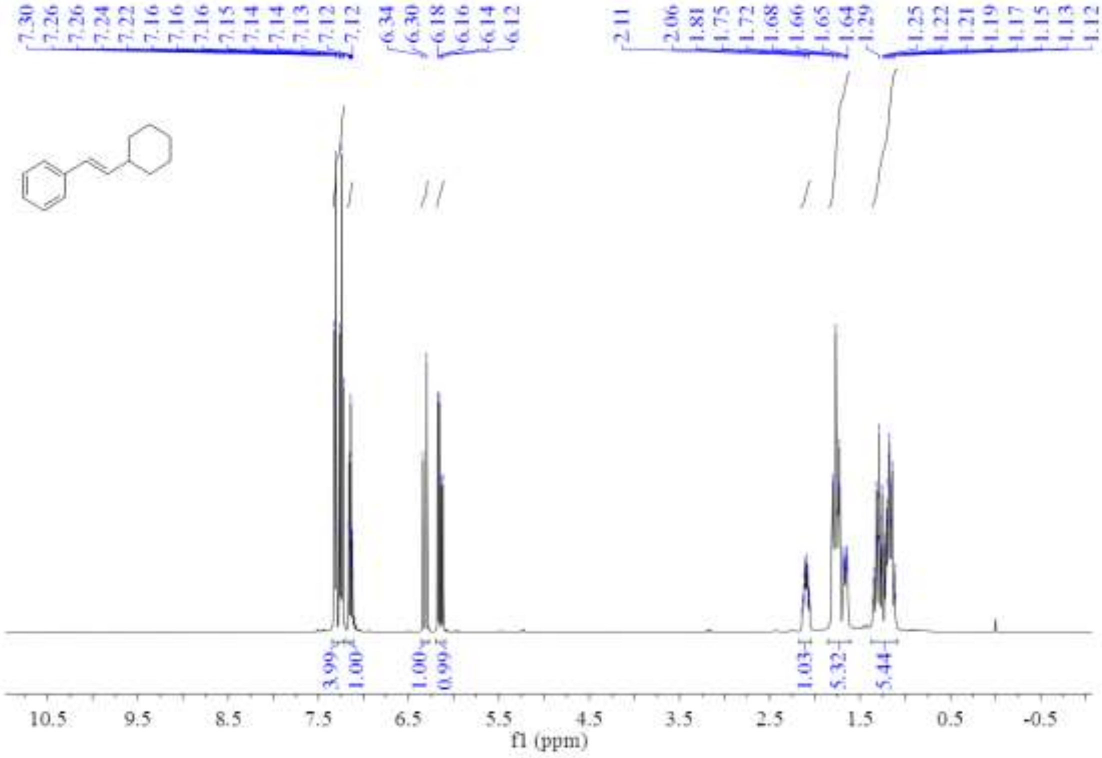

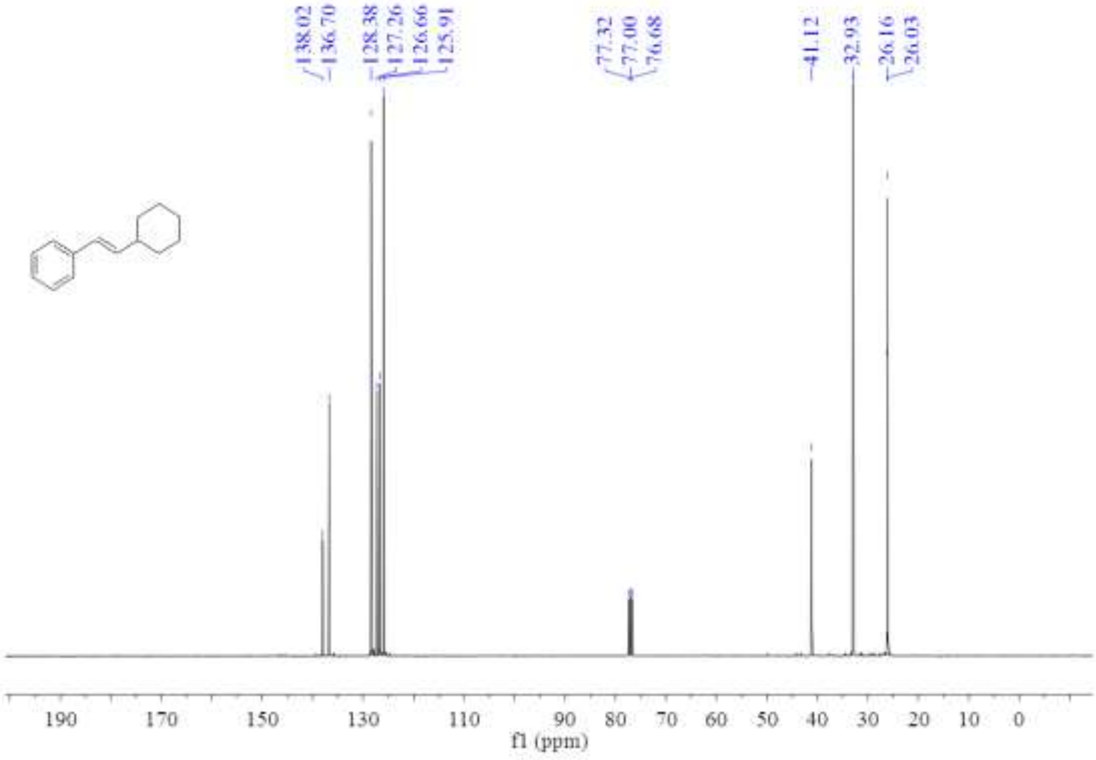

^{1}H- and ^{13}C NMR of 3b

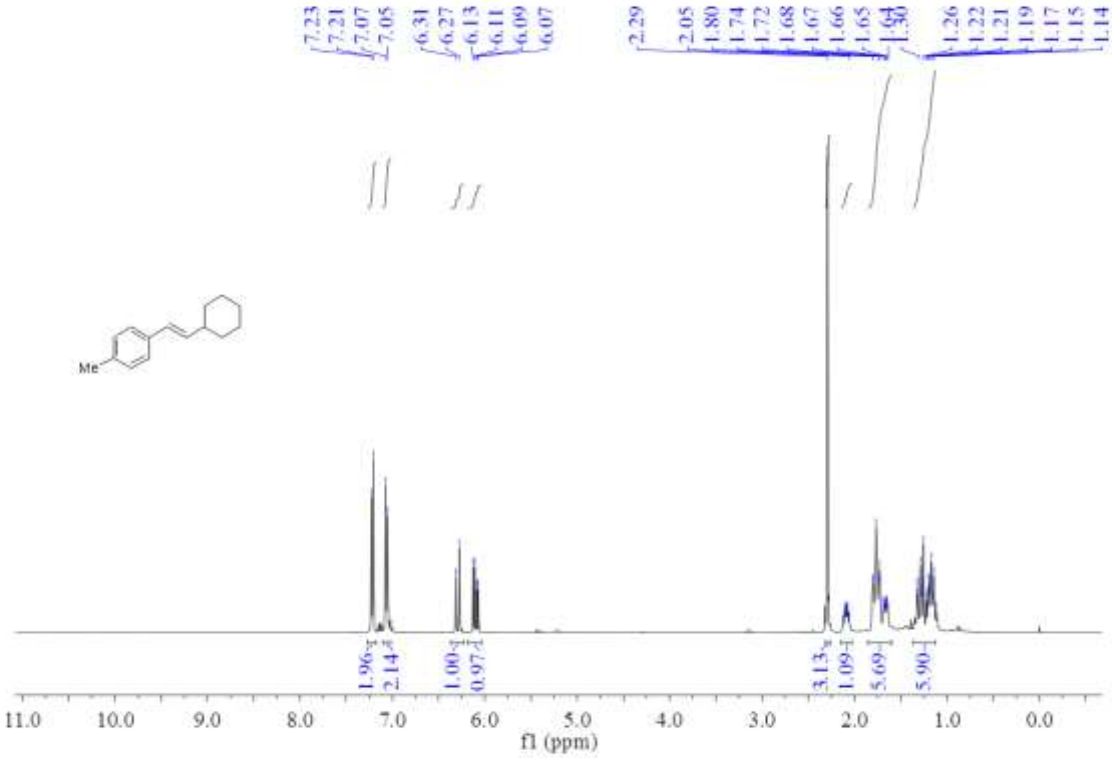

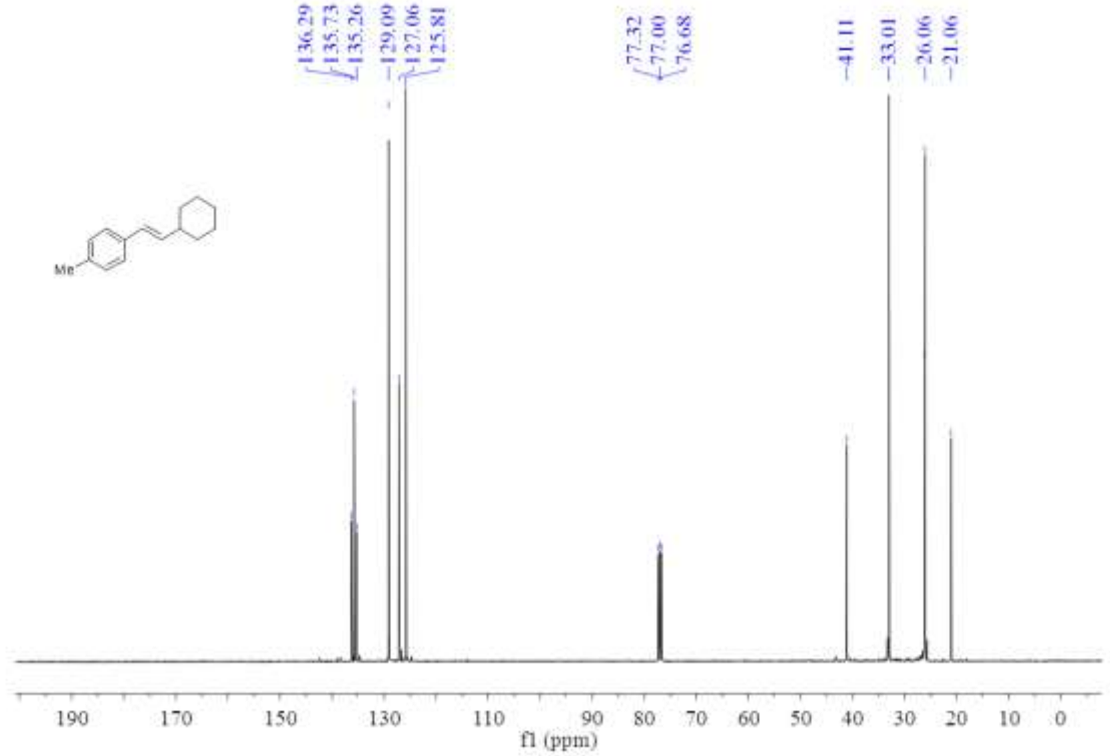

^{1}H- and ^{13}C NMR of 3c

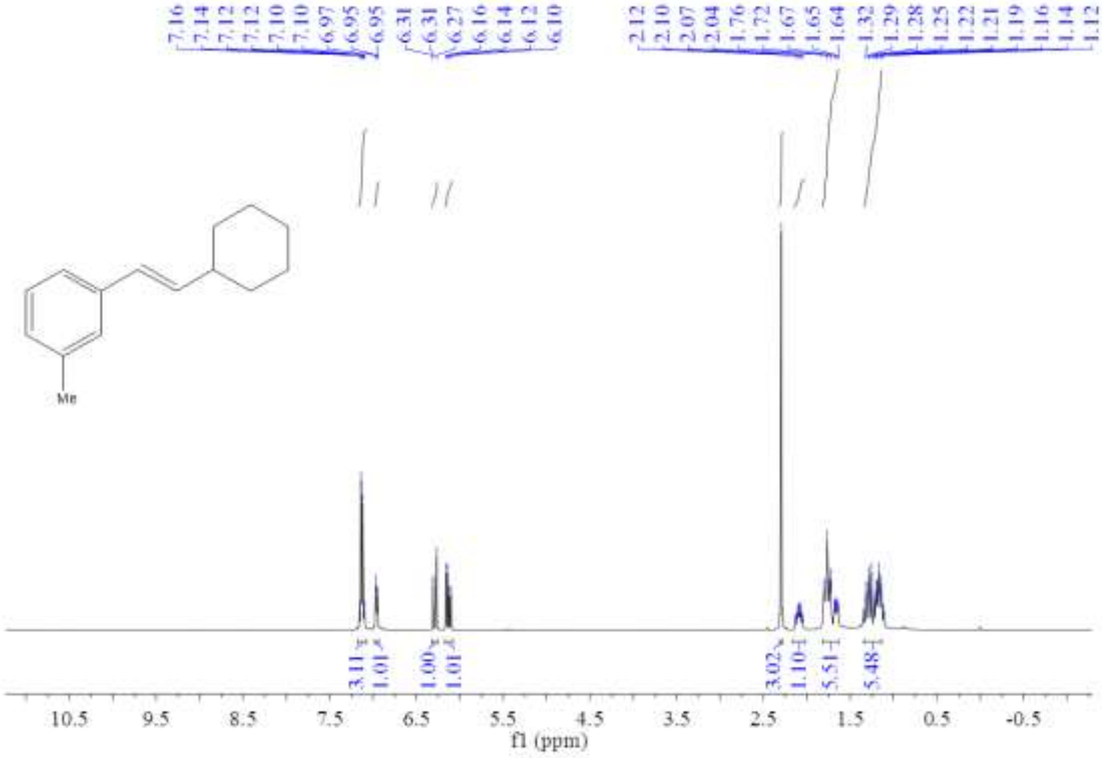

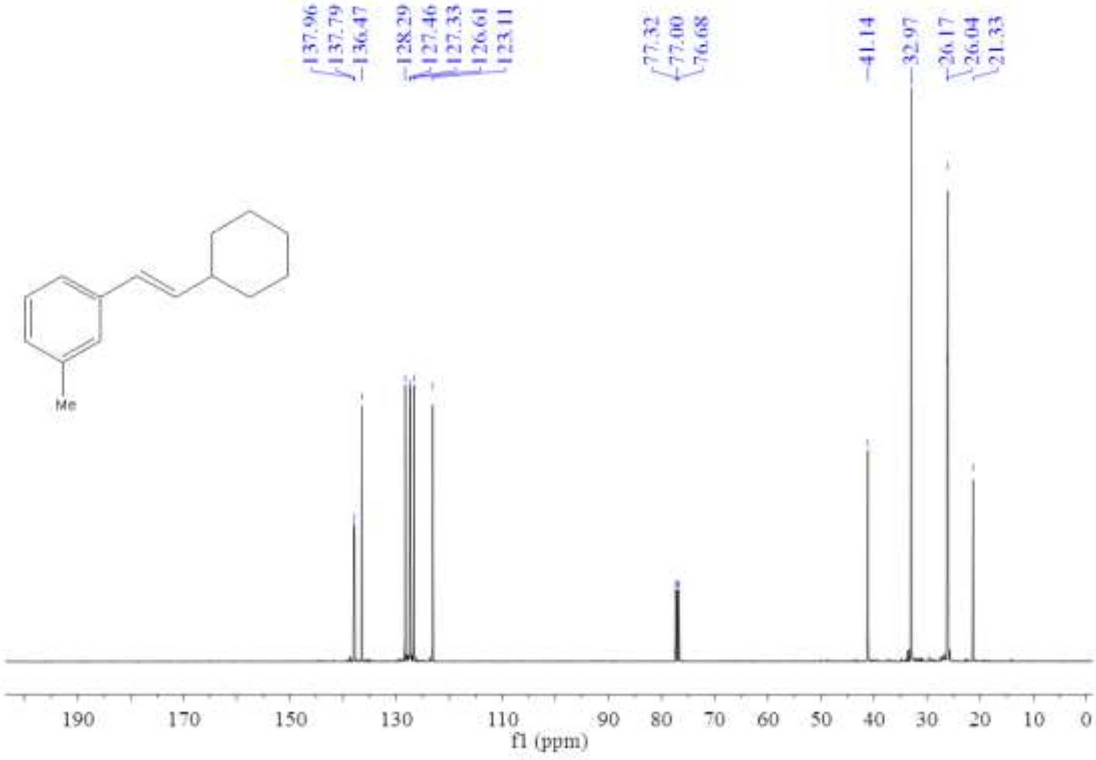

^{1}H- and ^{13}C NMR of 3d

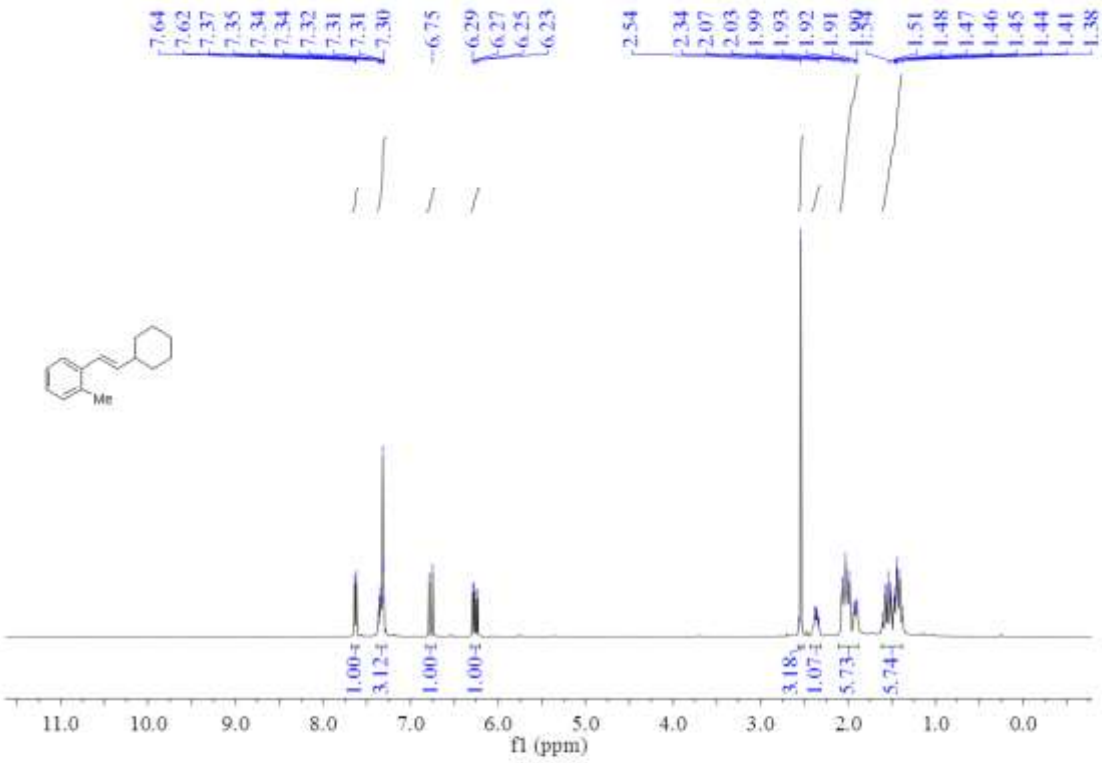

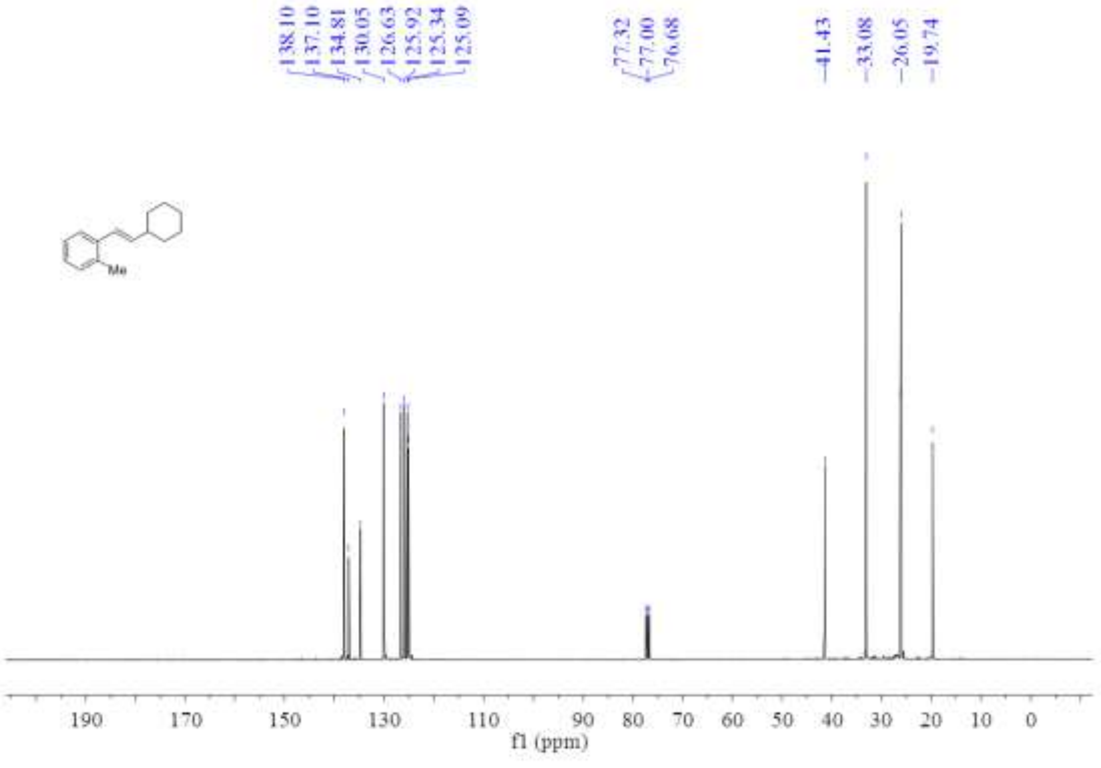

^{1}H- and ^{13}C NMR of 3e

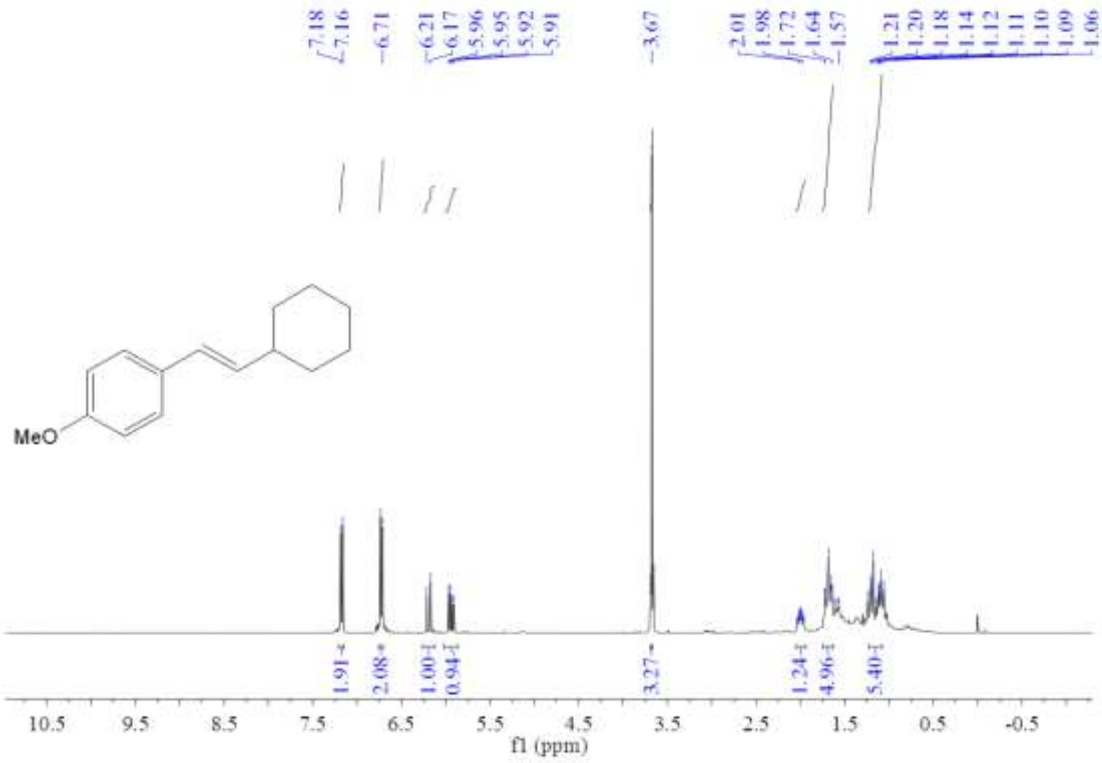

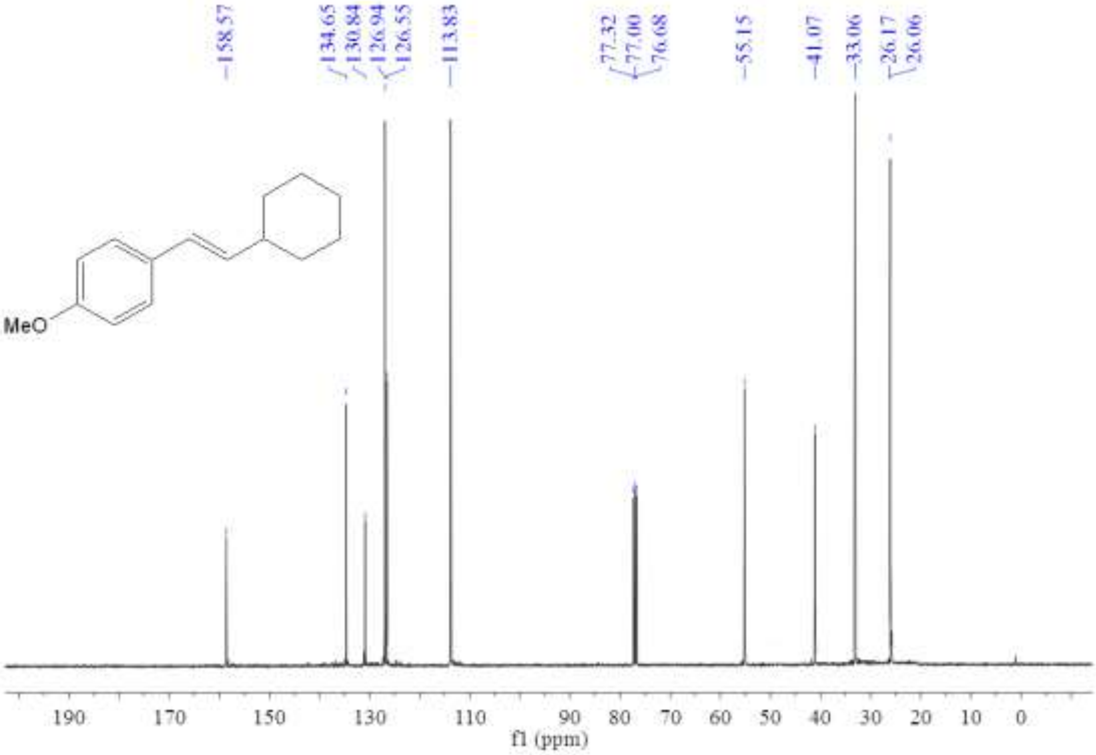

^{1}H- and ^{13}C NMR of 3f

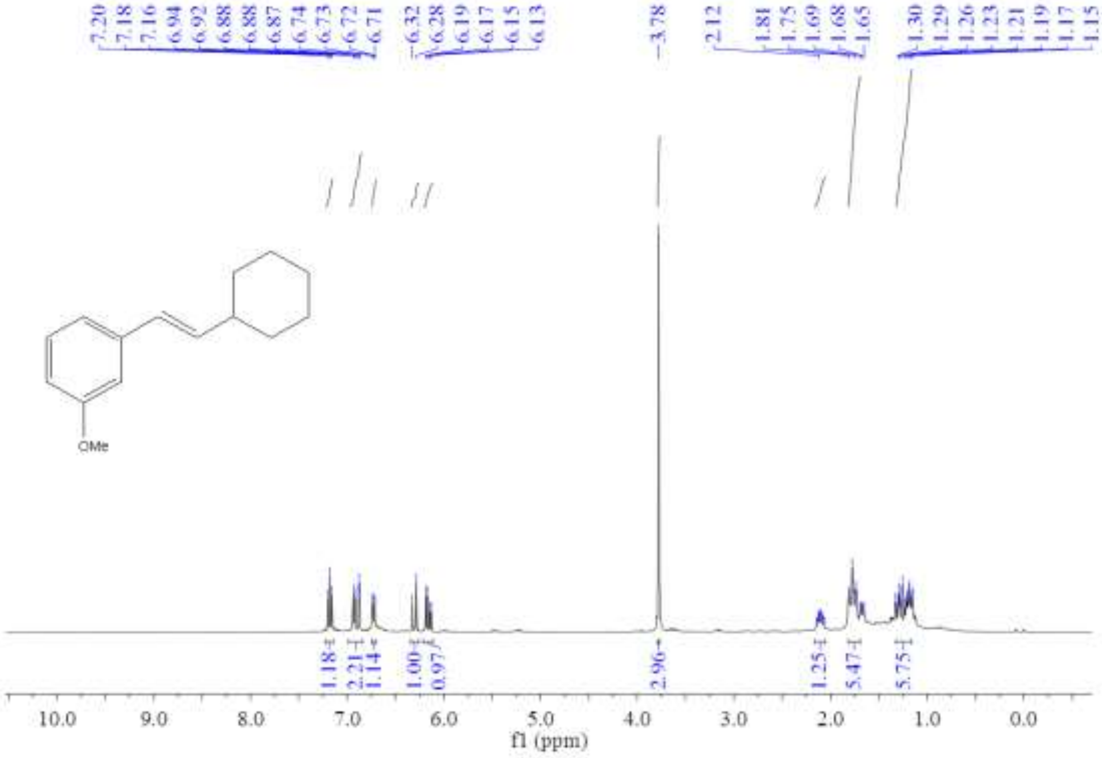

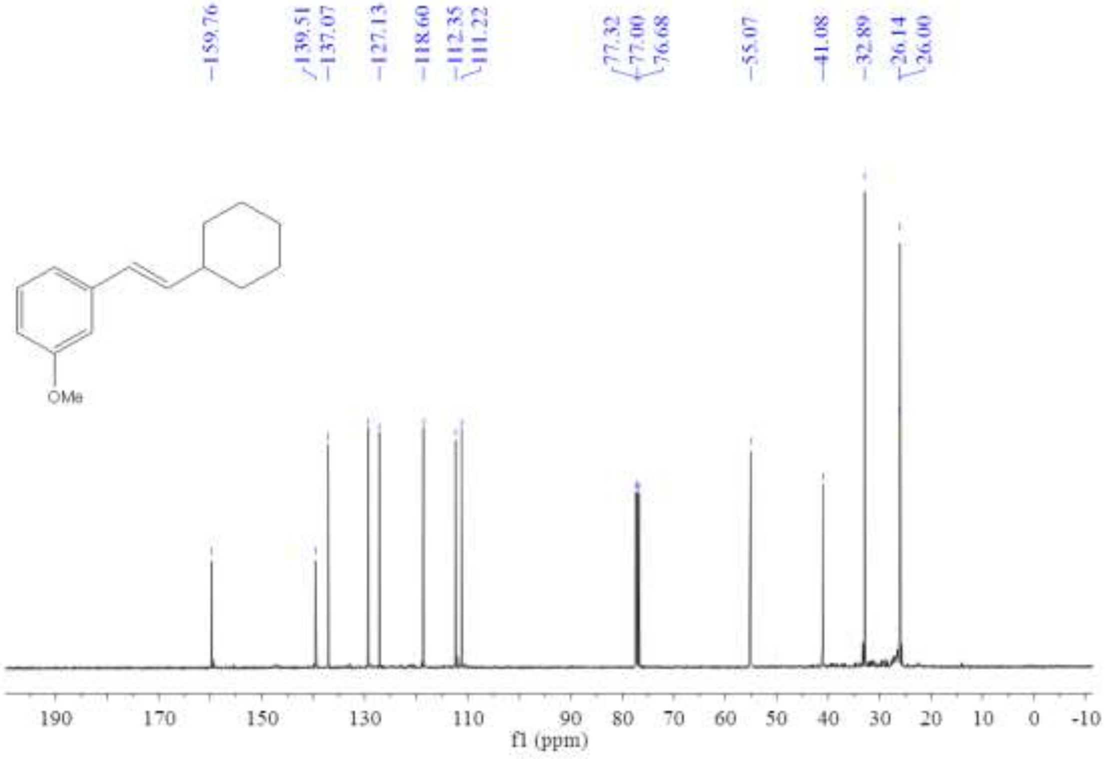

^{1}H- and ^{13}C NMR of 3g

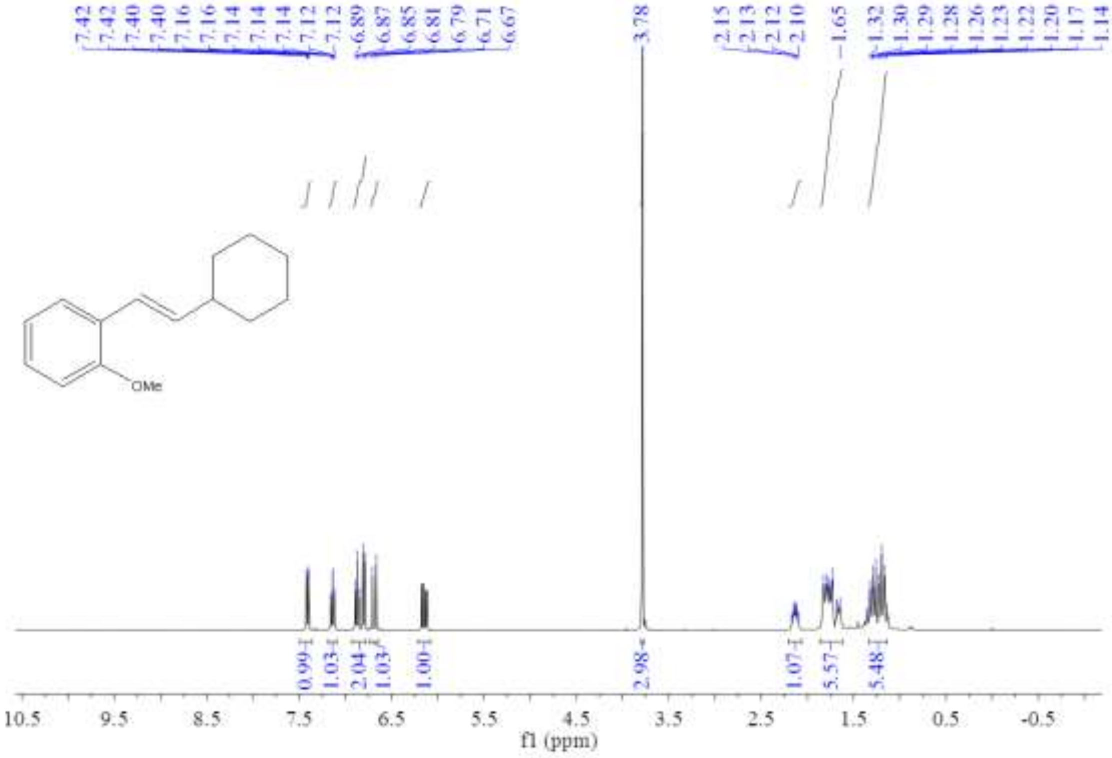

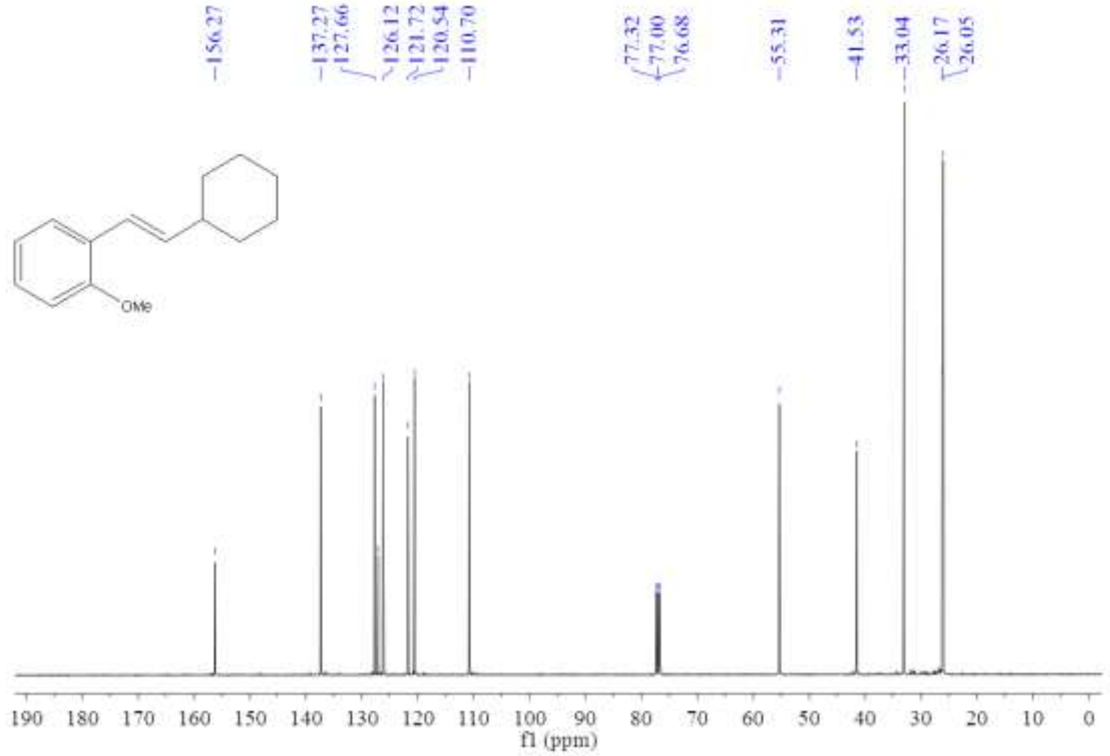

^{1}H- and ^{13}C NMR of 3h

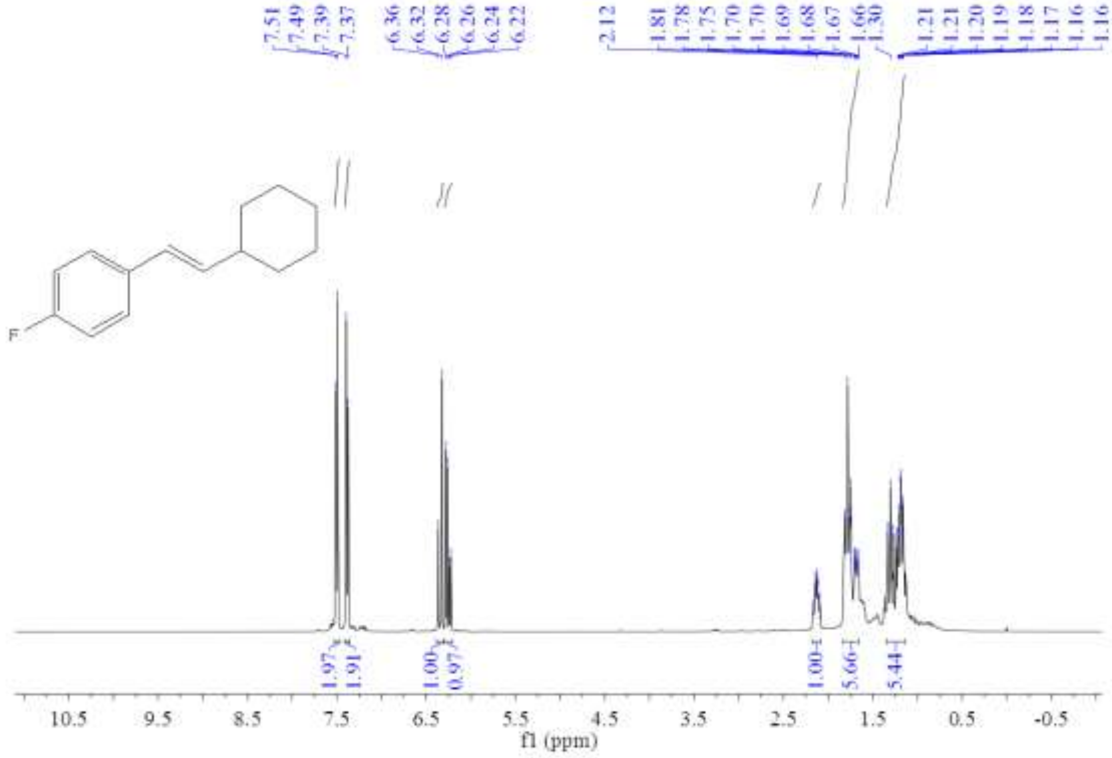

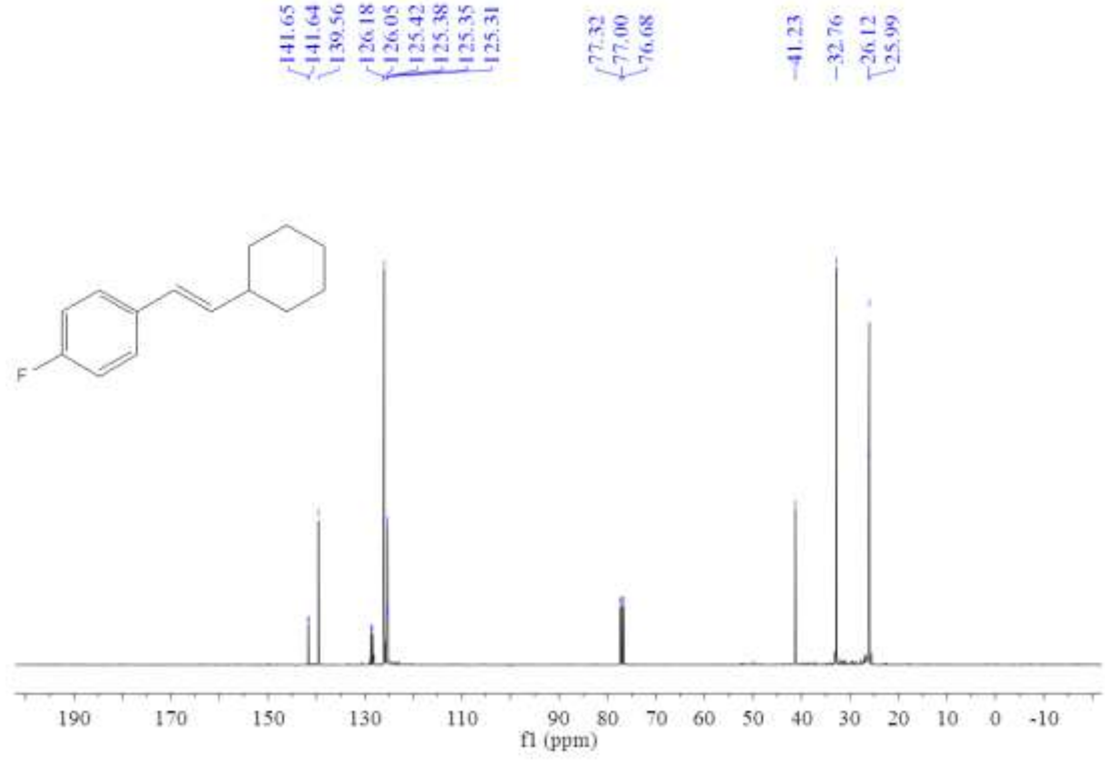

^{1}H- and ^{13}C NMR of 3i

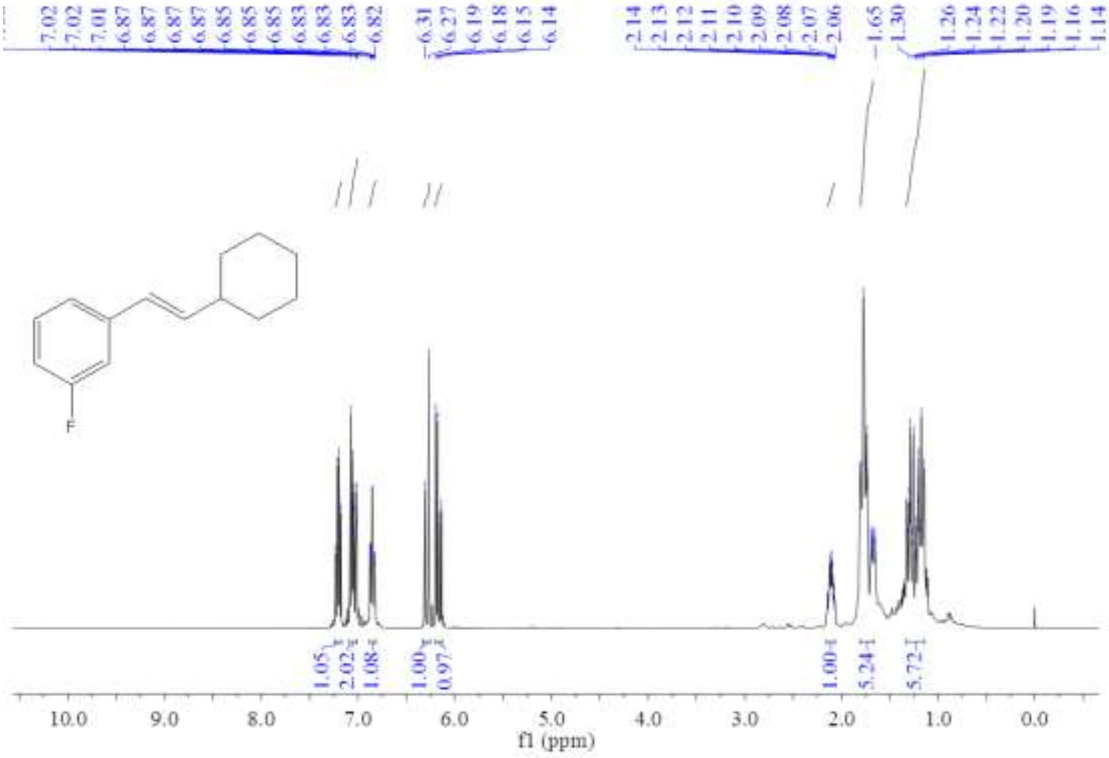

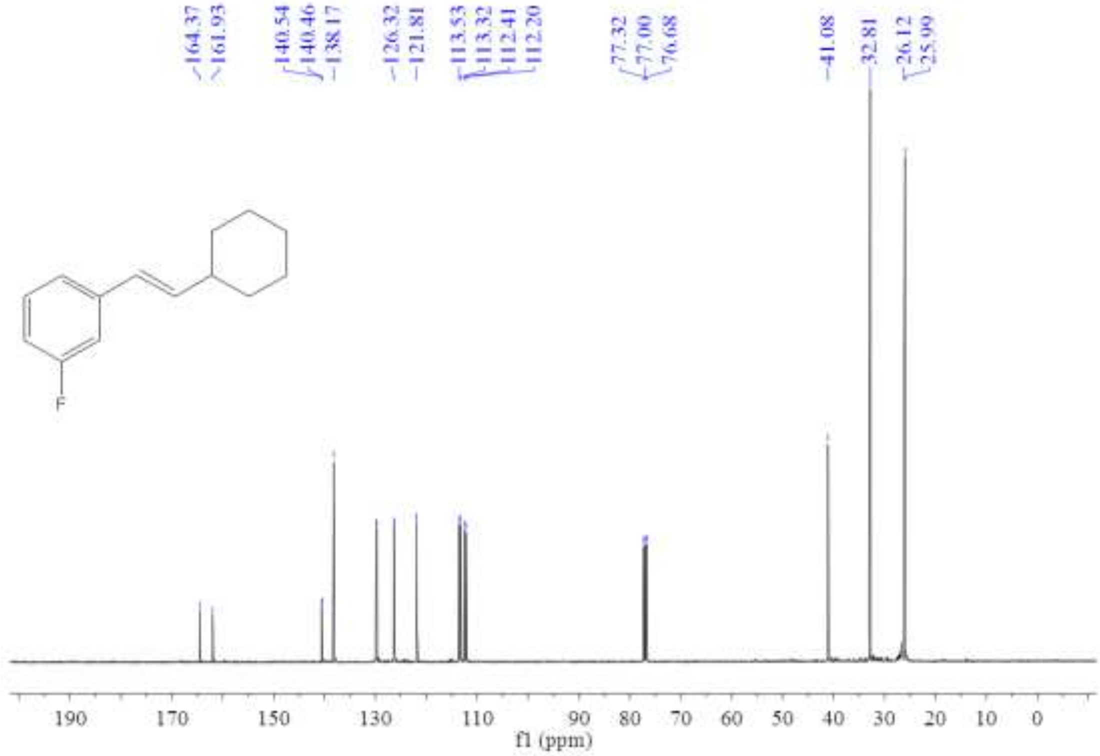

^{1}H- and ^{13}C NMR of 3j

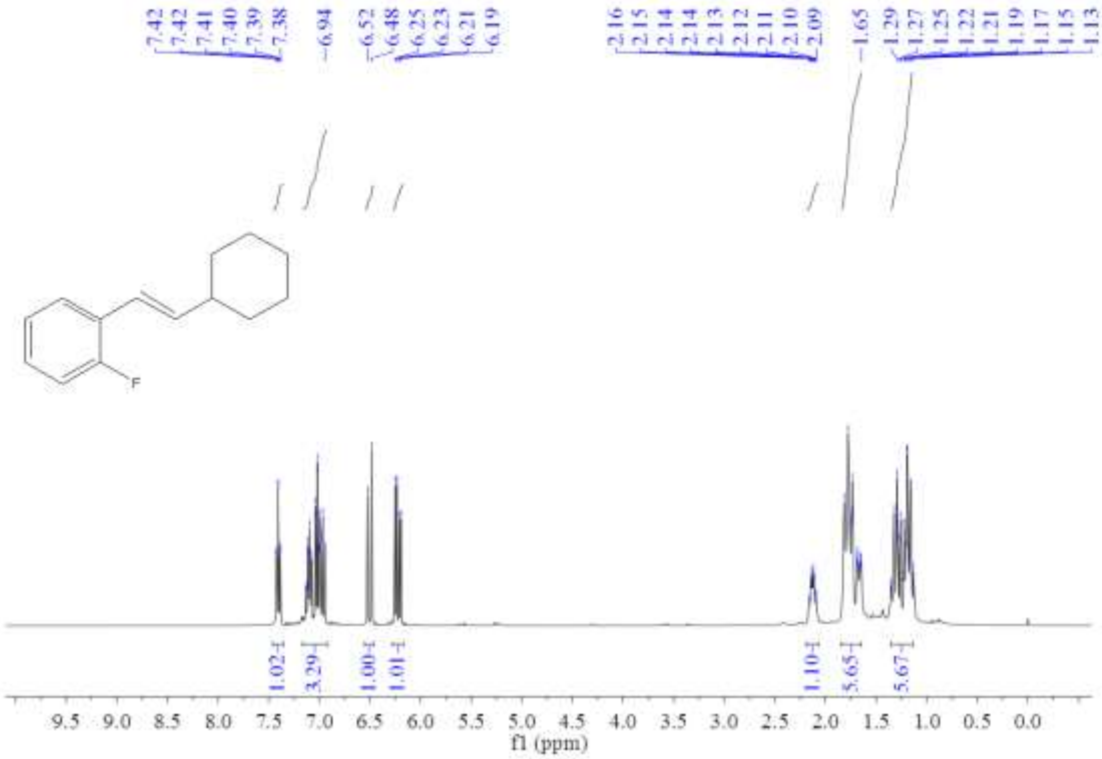

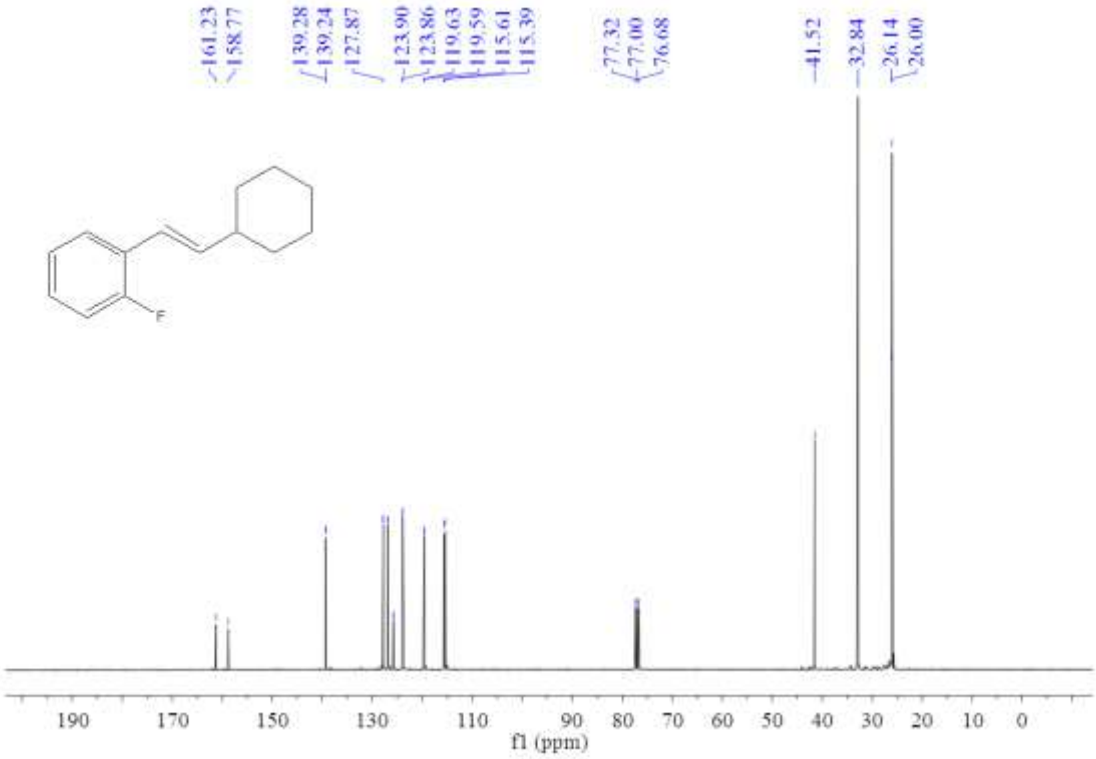

^{1}H- and ^{13}C NMR of 3k

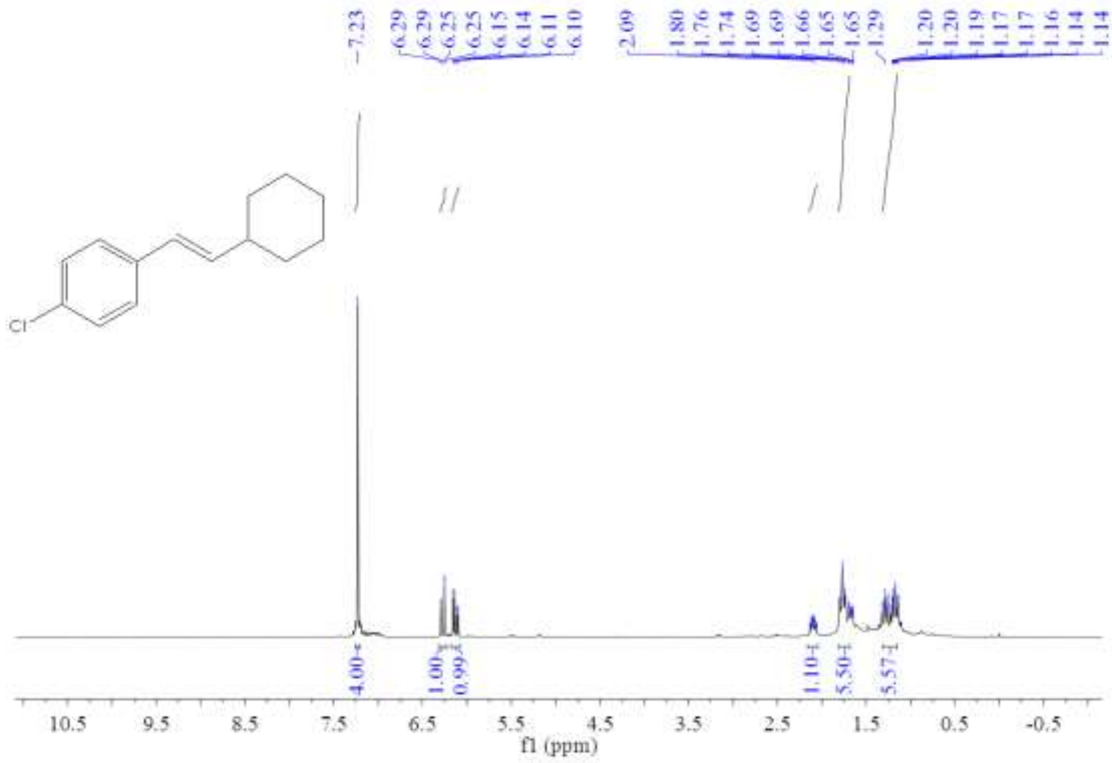

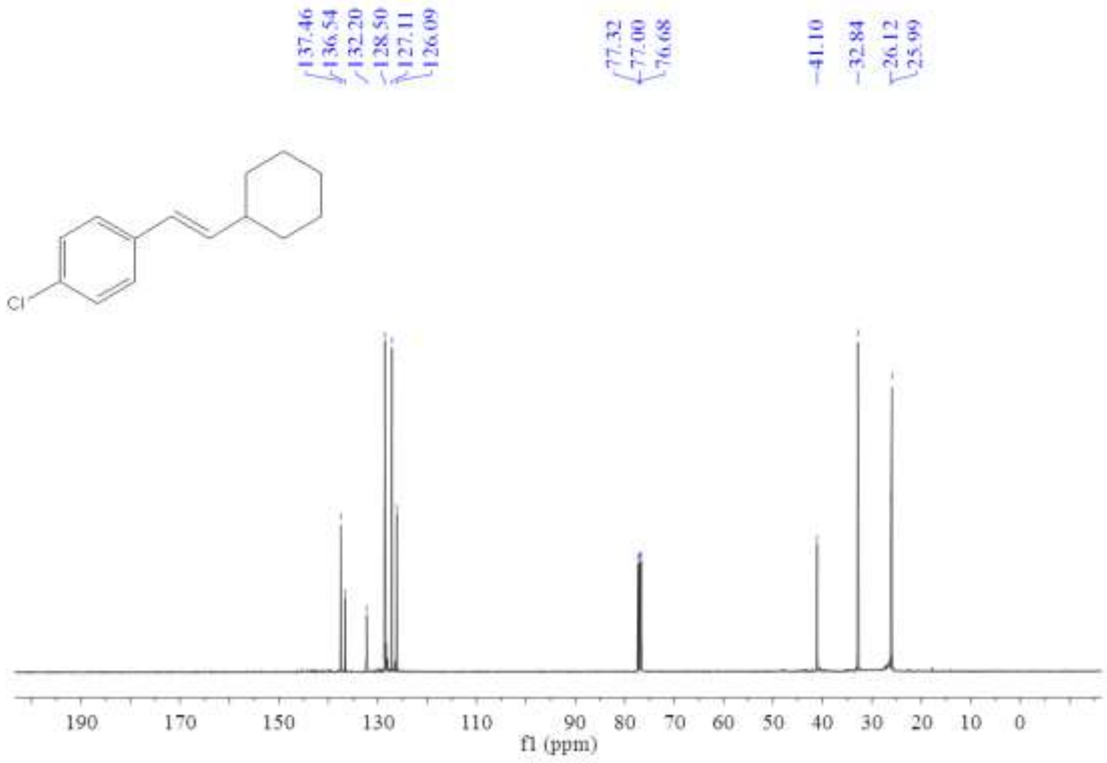

^{1}H- and ^{13}C NMR of 3l

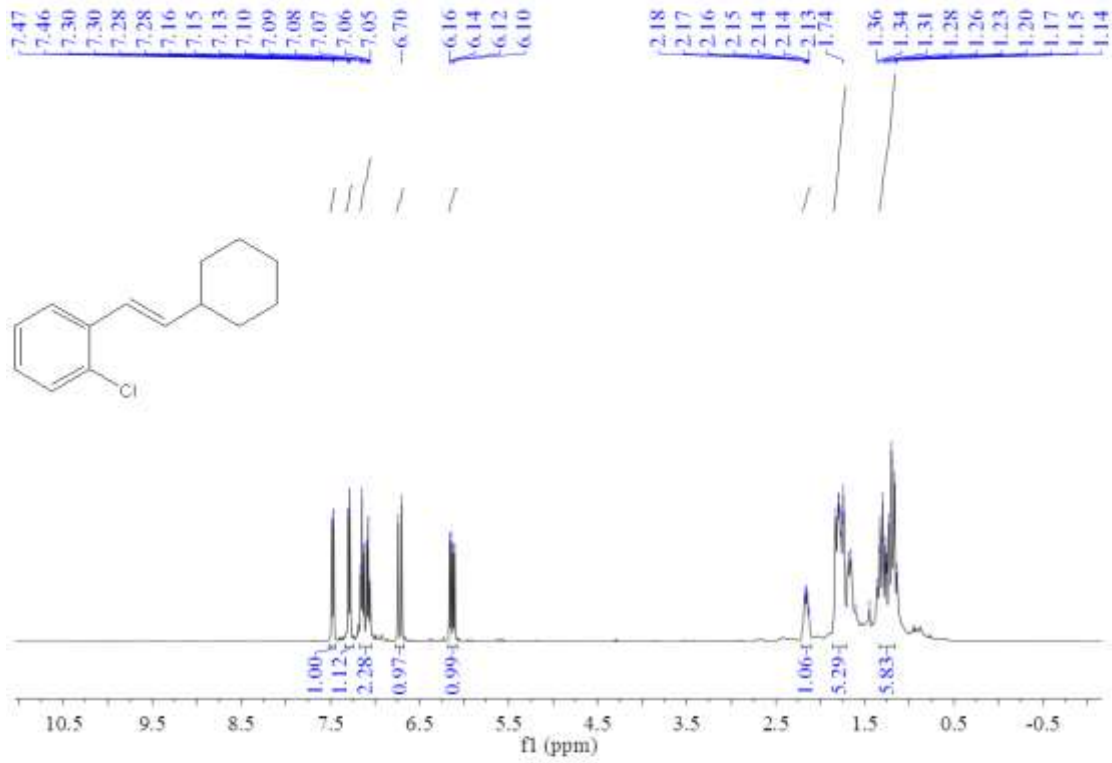

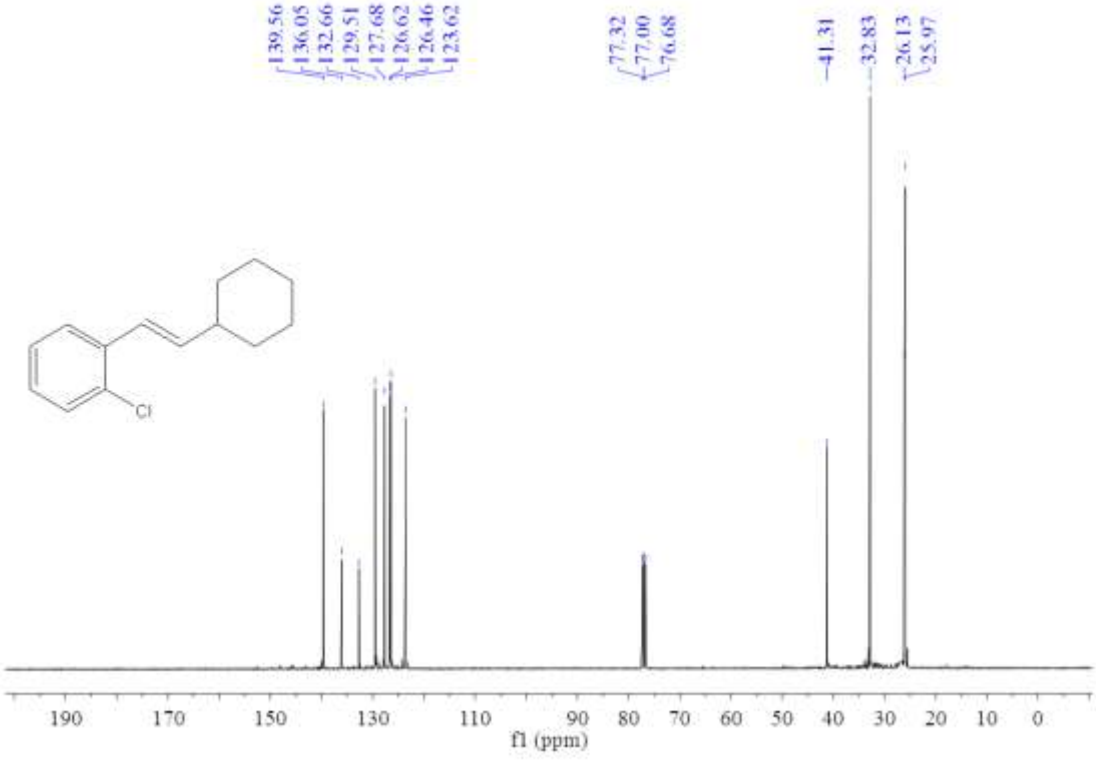

^{1}H- and ^{13}C NMR of 3m

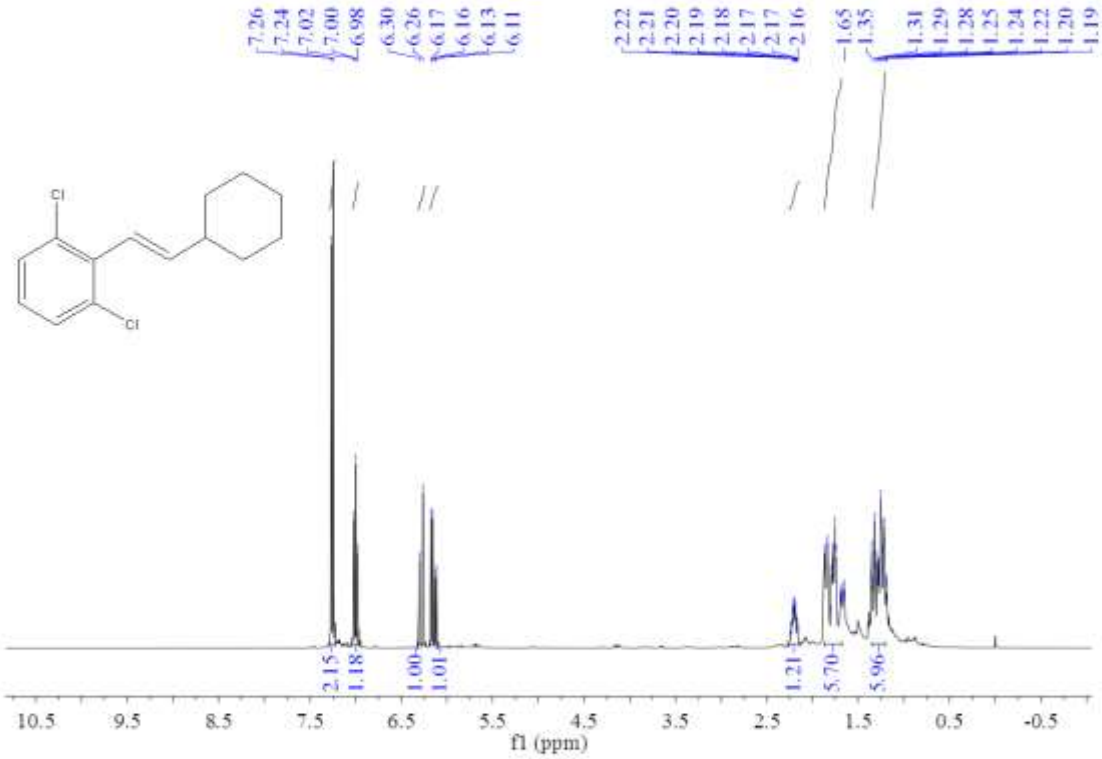

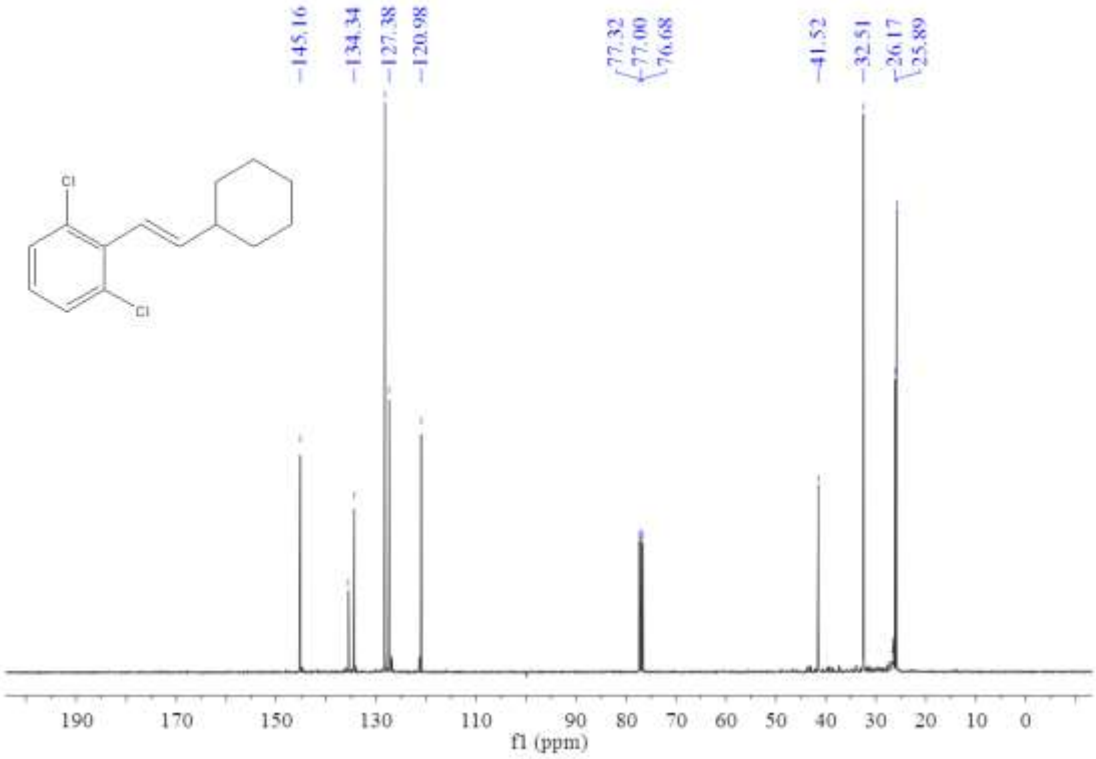

^{1}H- and ^{13}C NMR of 3n

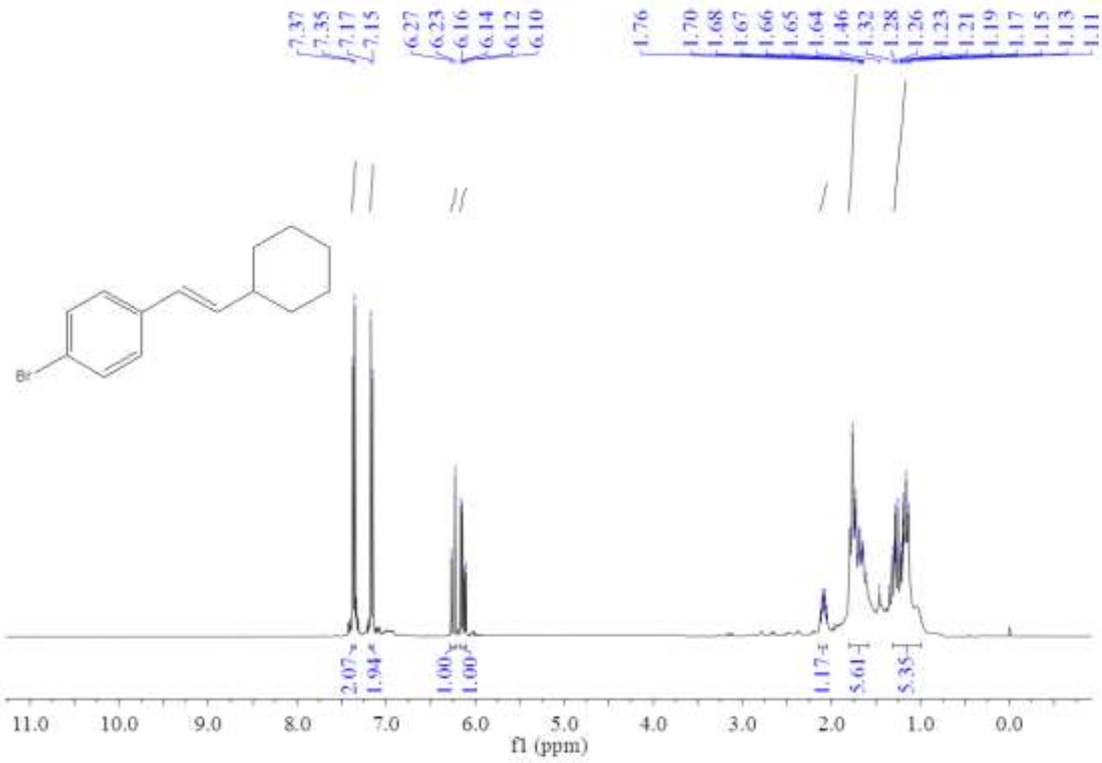

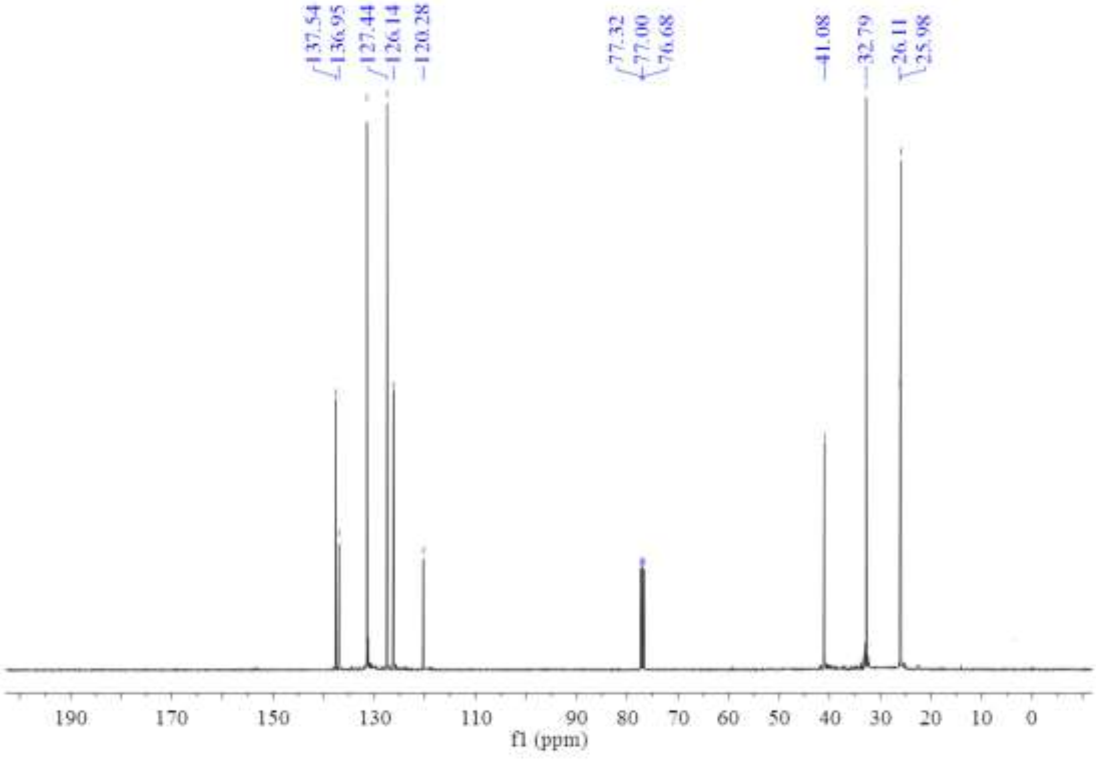

^{1}H- and ^{13}C NMR of 3o

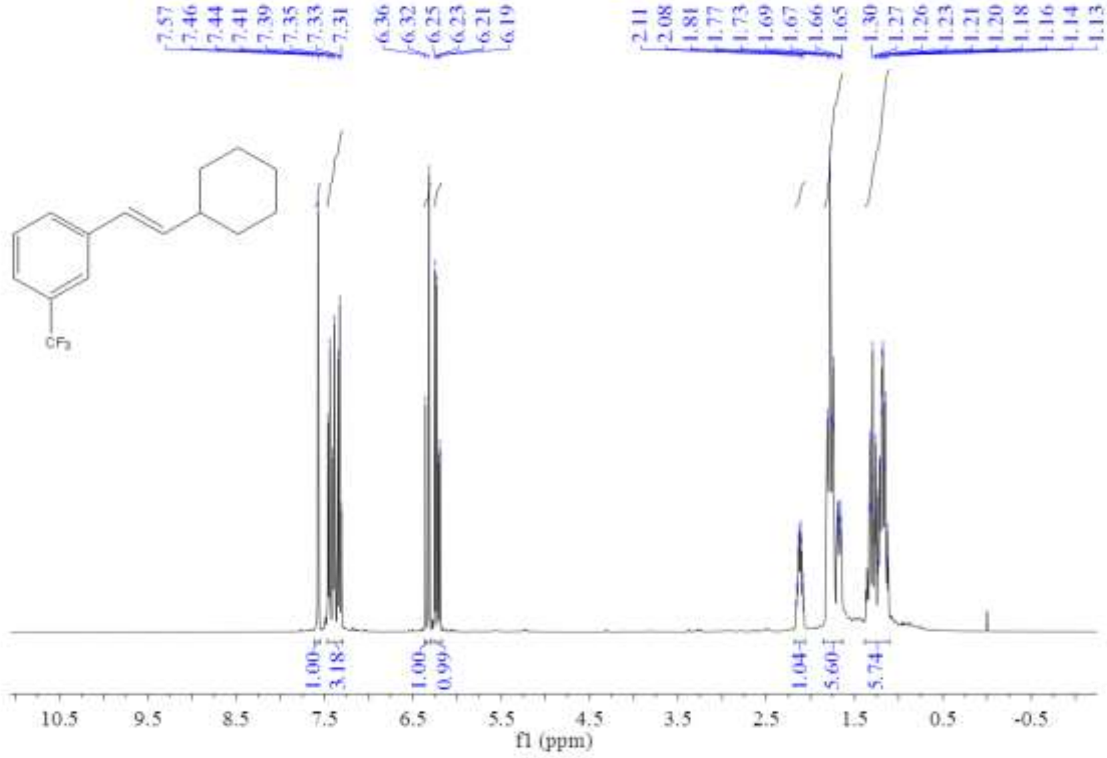

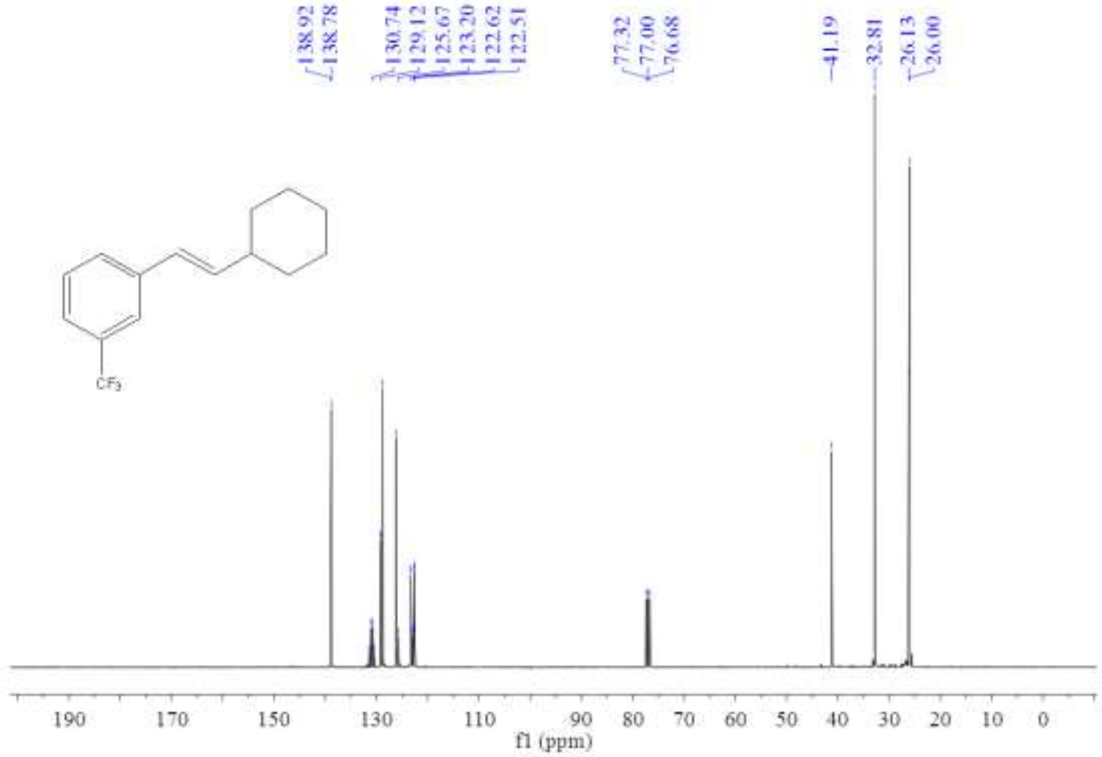

^{1}H- and ^{13}C NMR of 3p

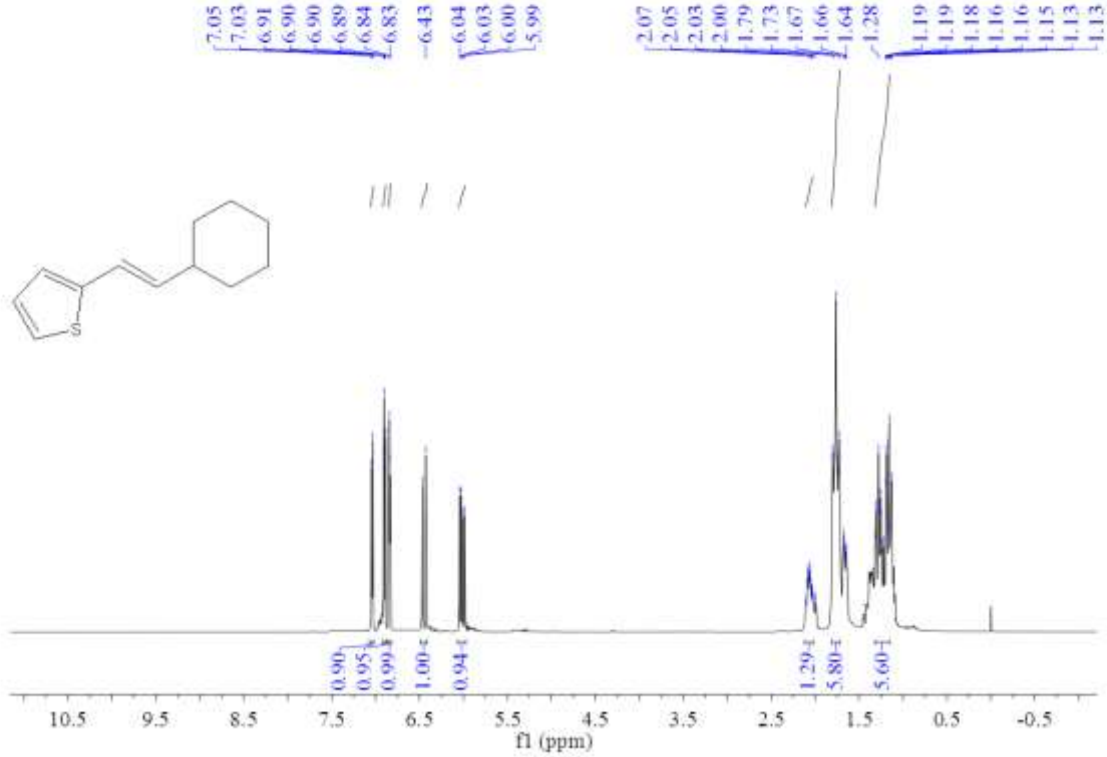

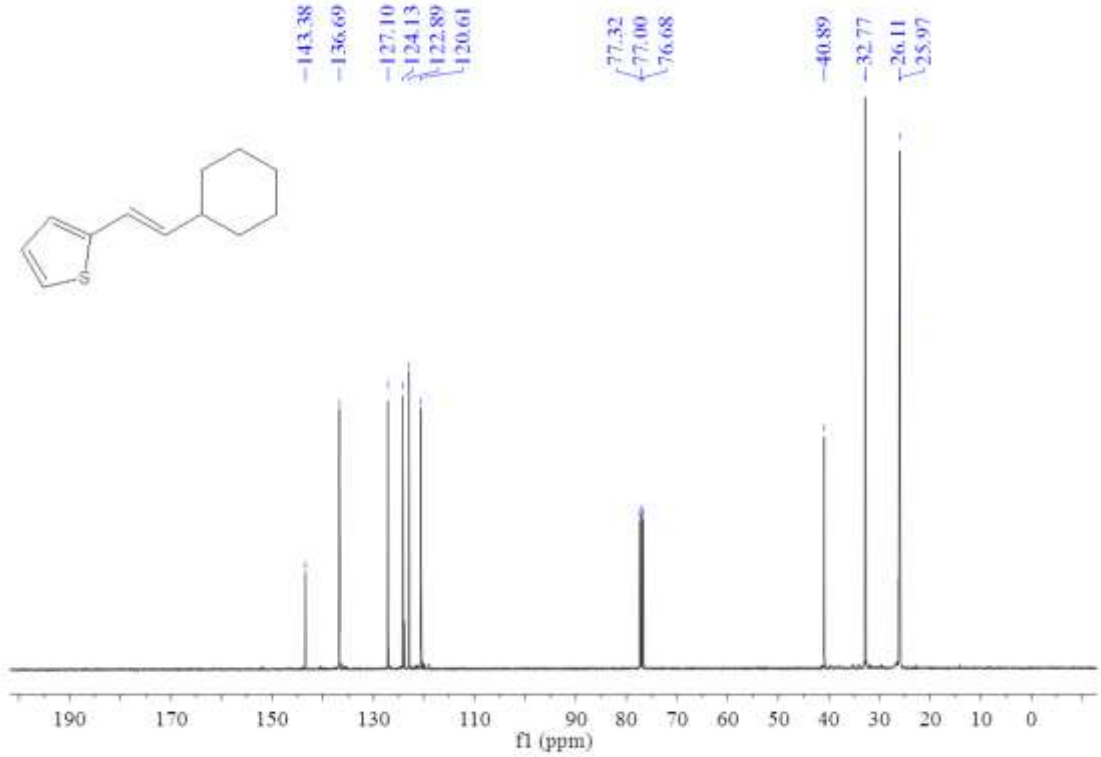

^{1}H- and ^{13}C NMR of 4a

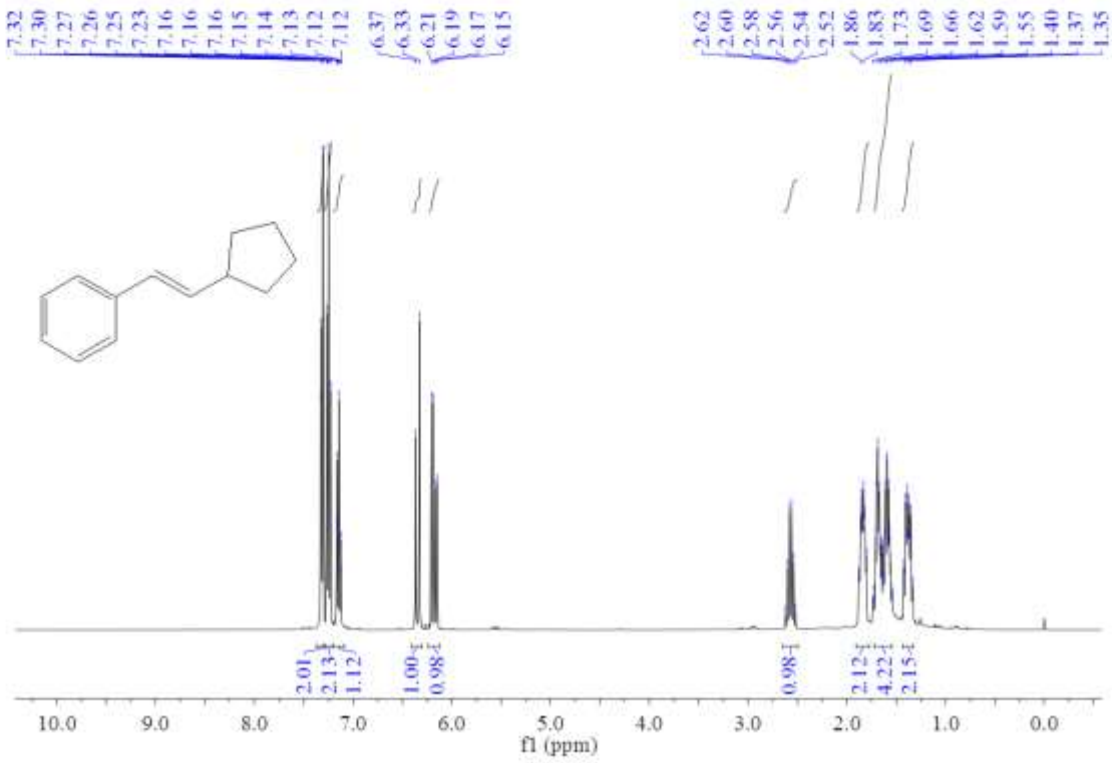

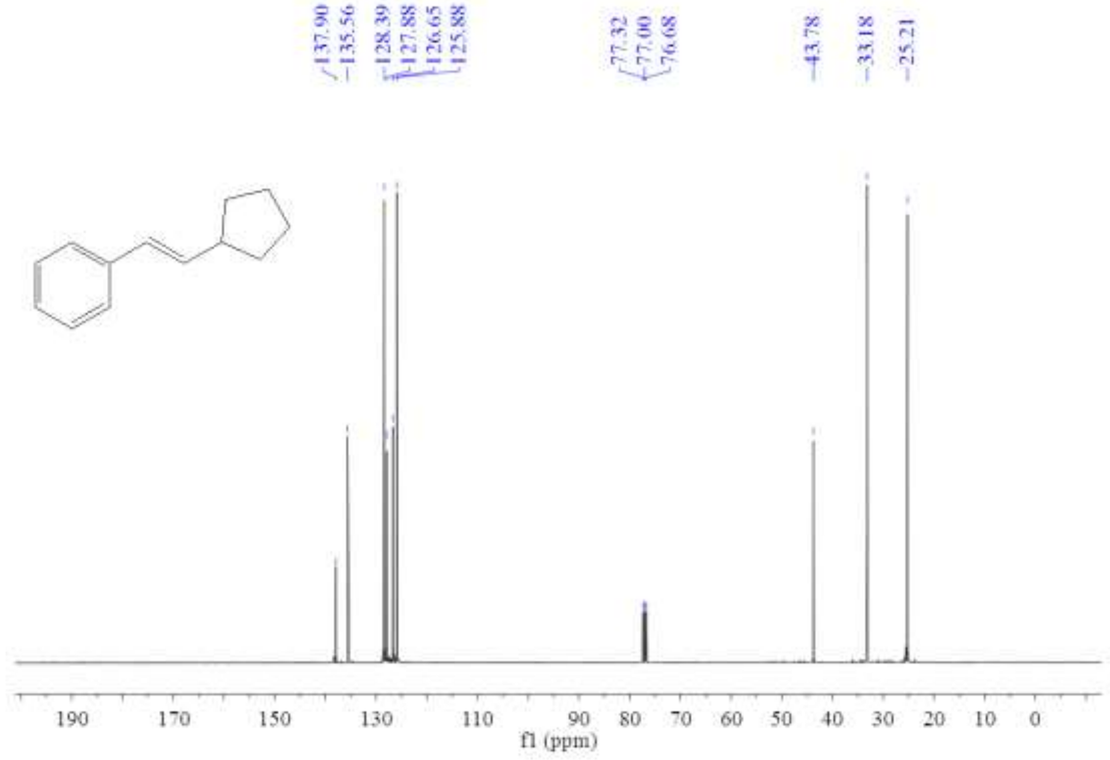

^{1}H- and ^{13}C NMR of 4b

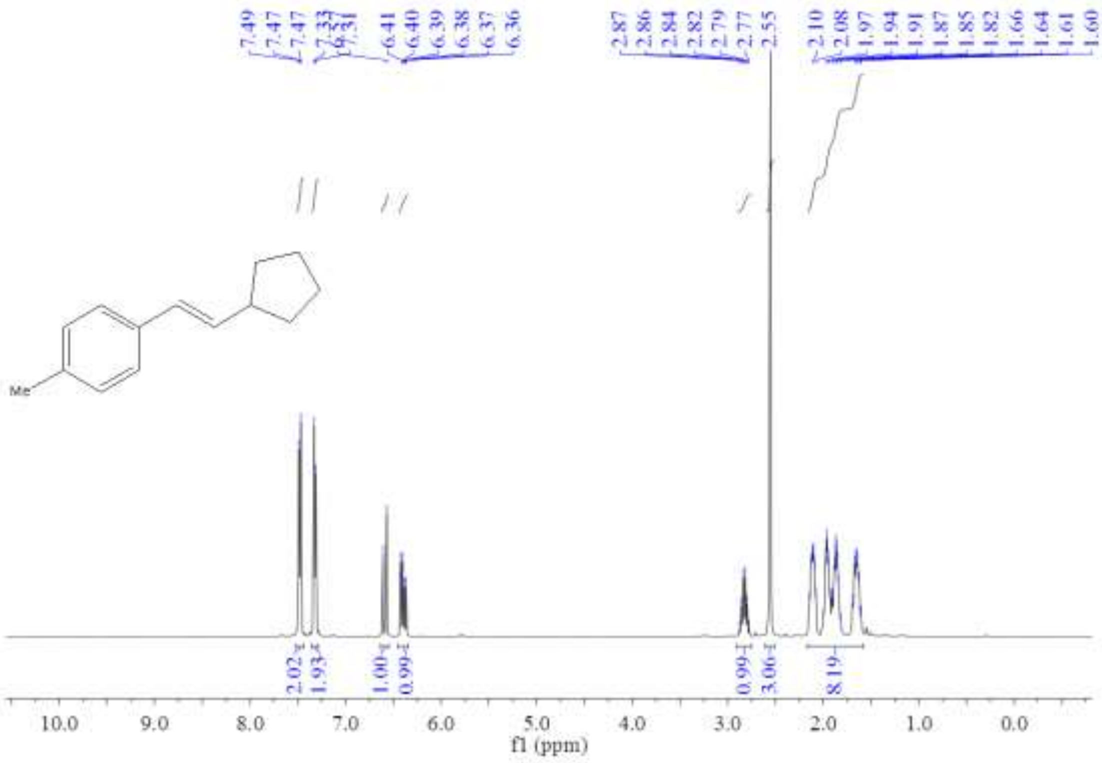

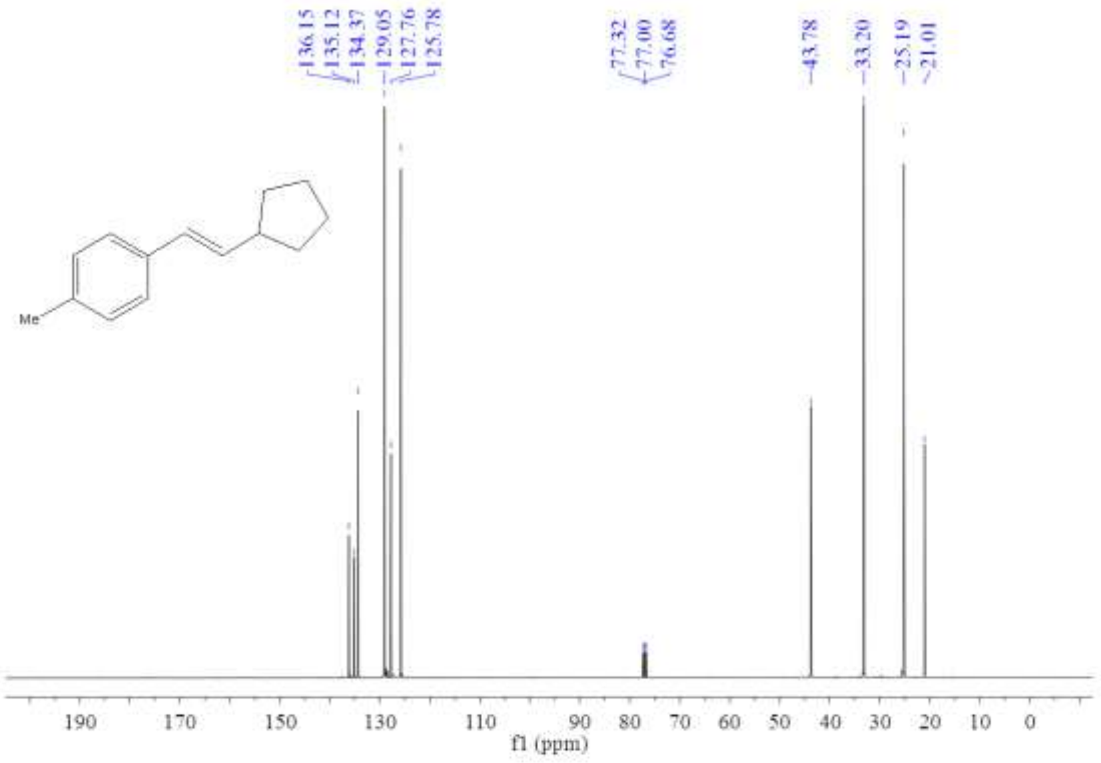

^{1}H- and ^{13}C NMR of 4c

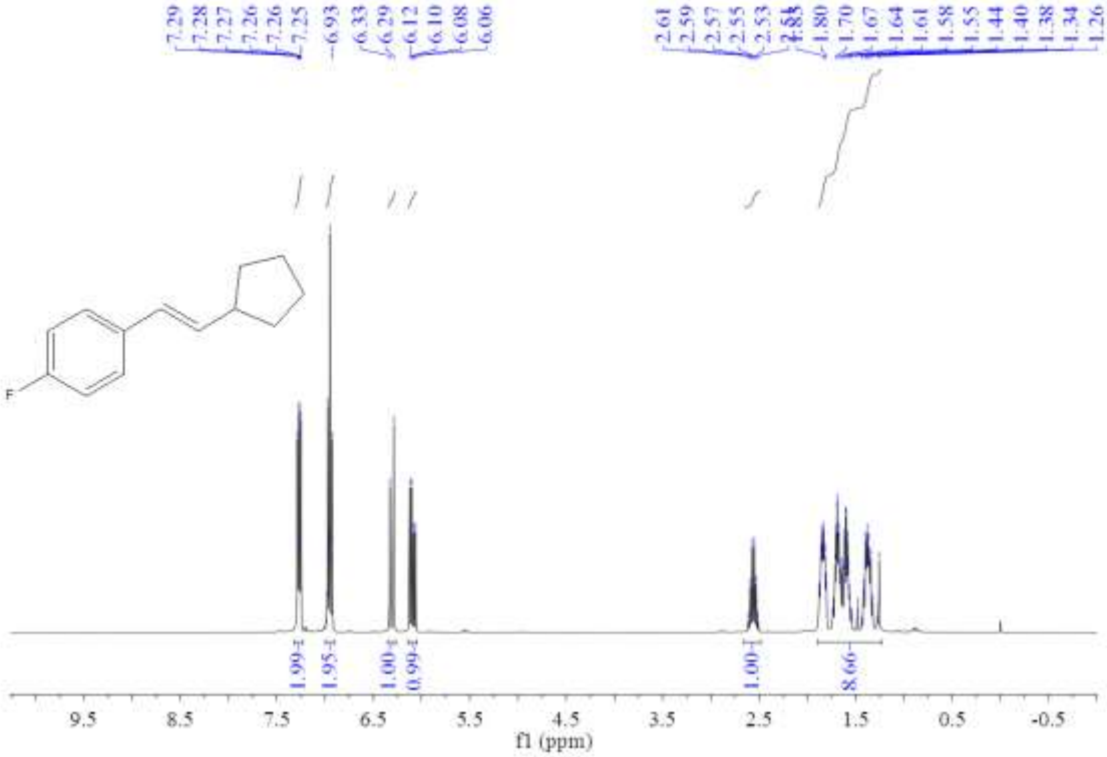

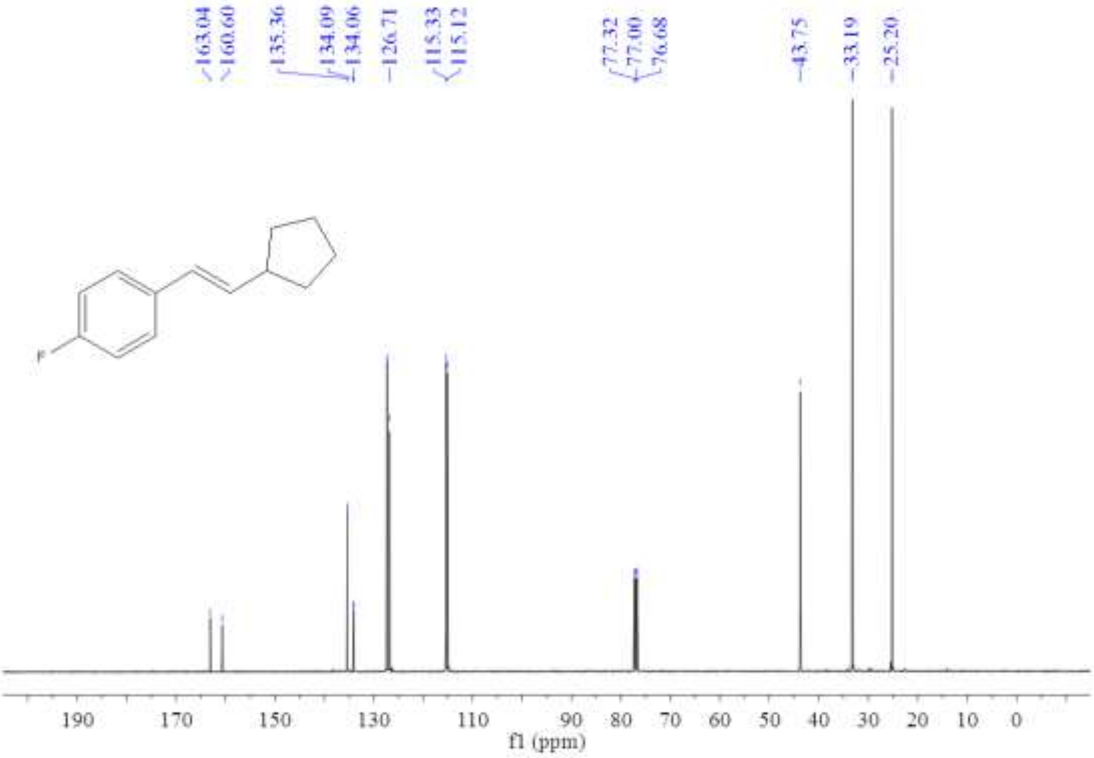

^{1}H- and ^{13}C NMR of 4d

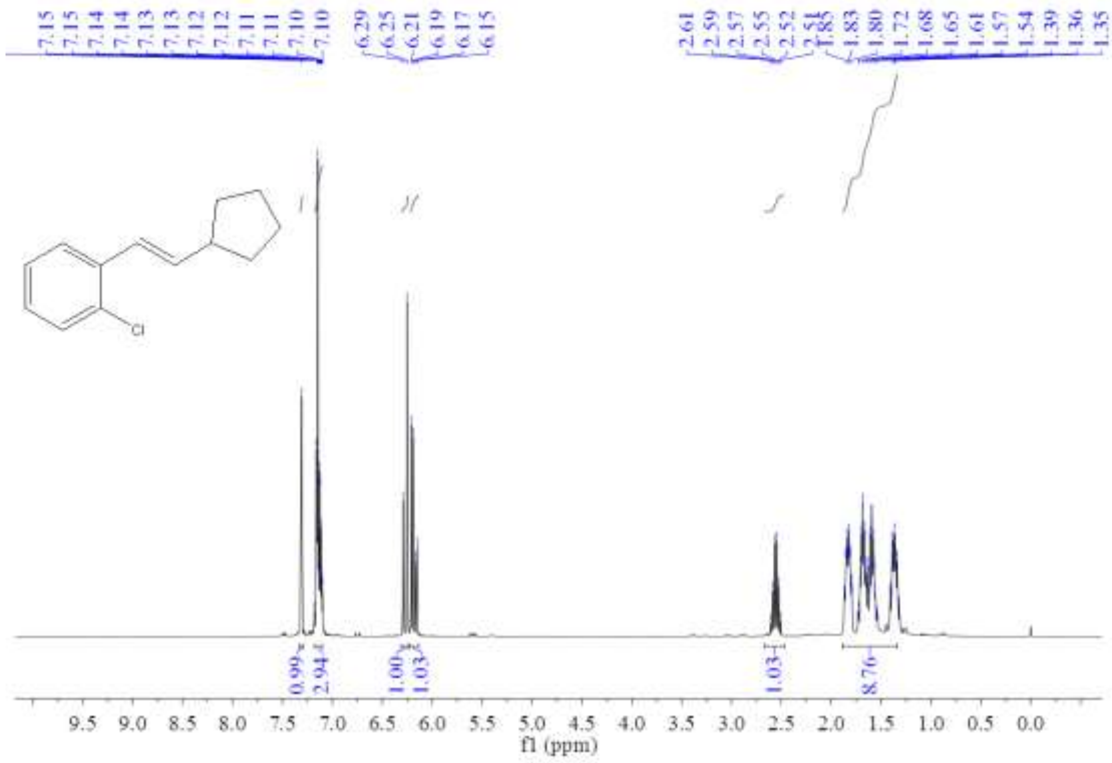

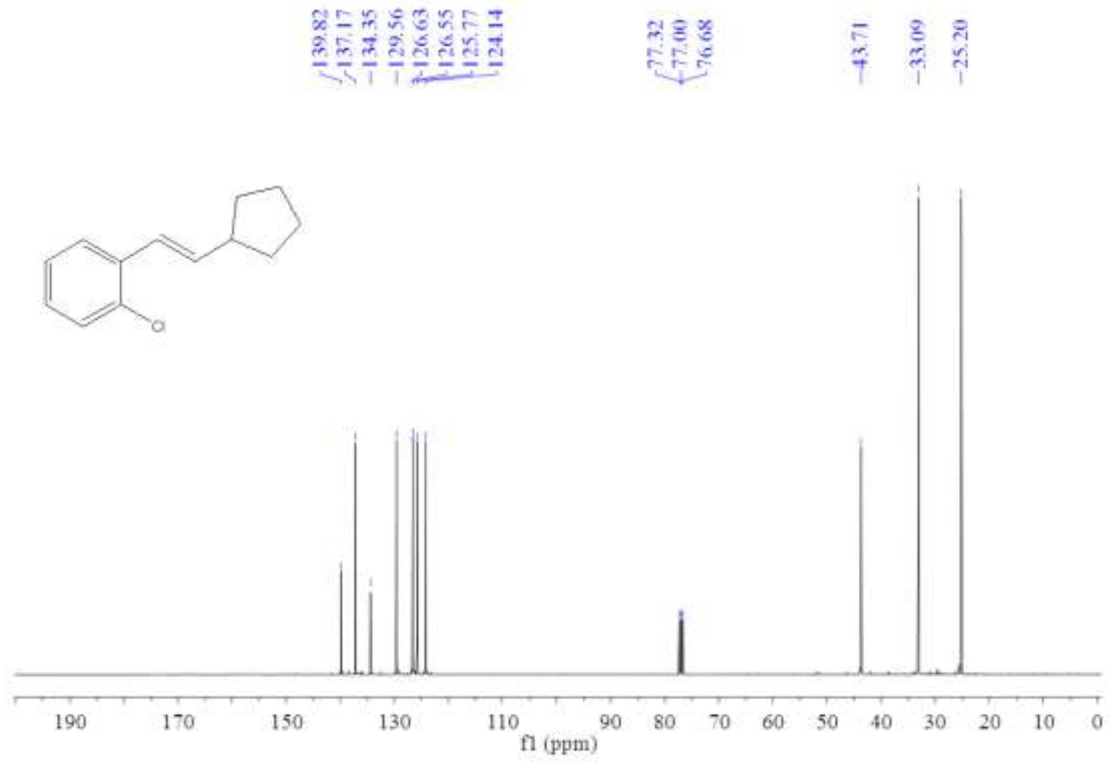

^{1}H- and ^{13}C NMR of 4e

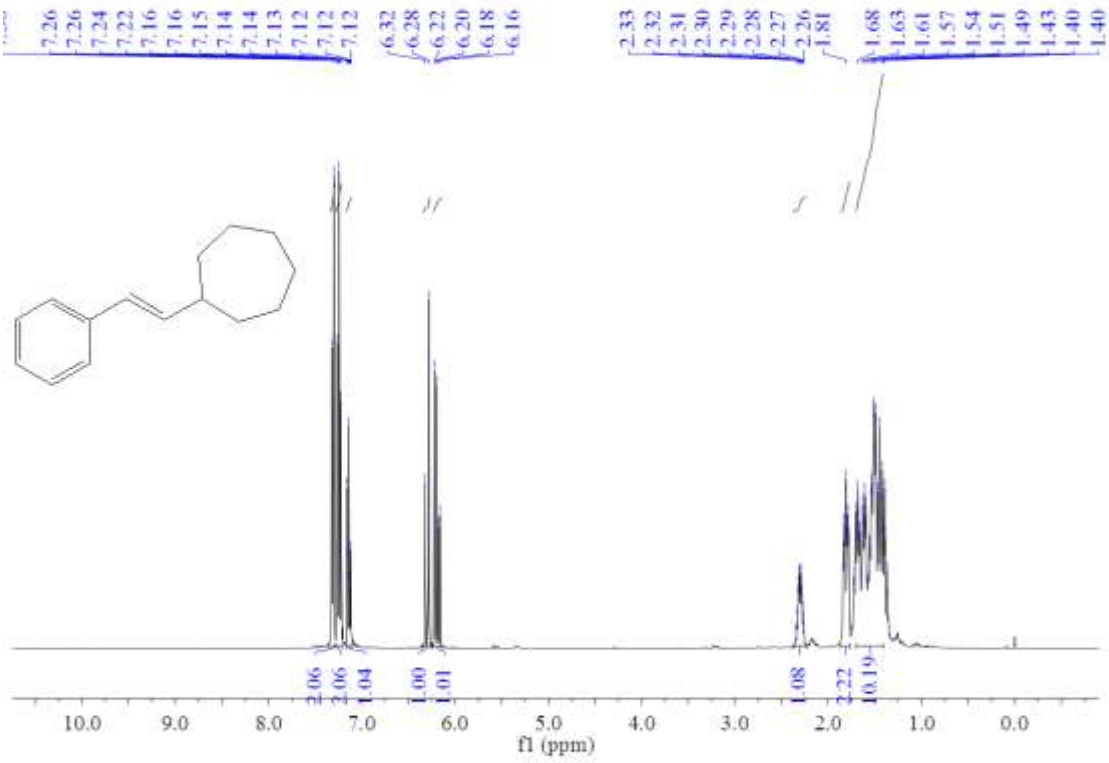

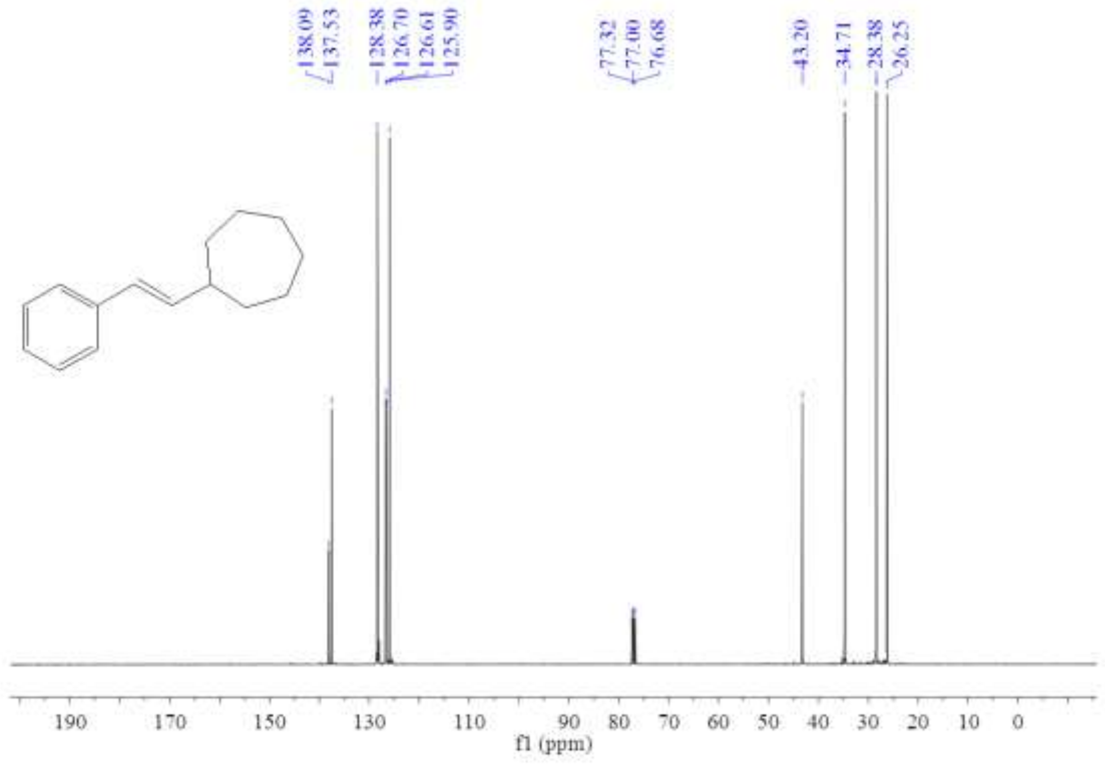

^{1}H- and ^{13}C NMR of 4f

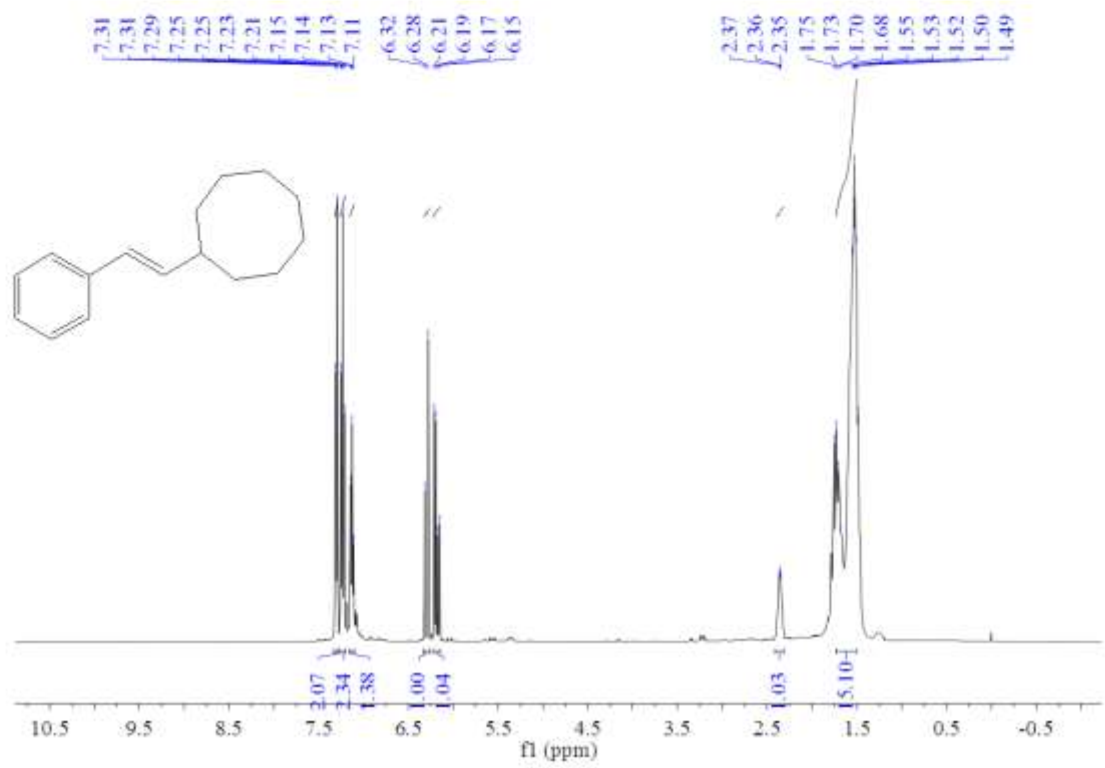

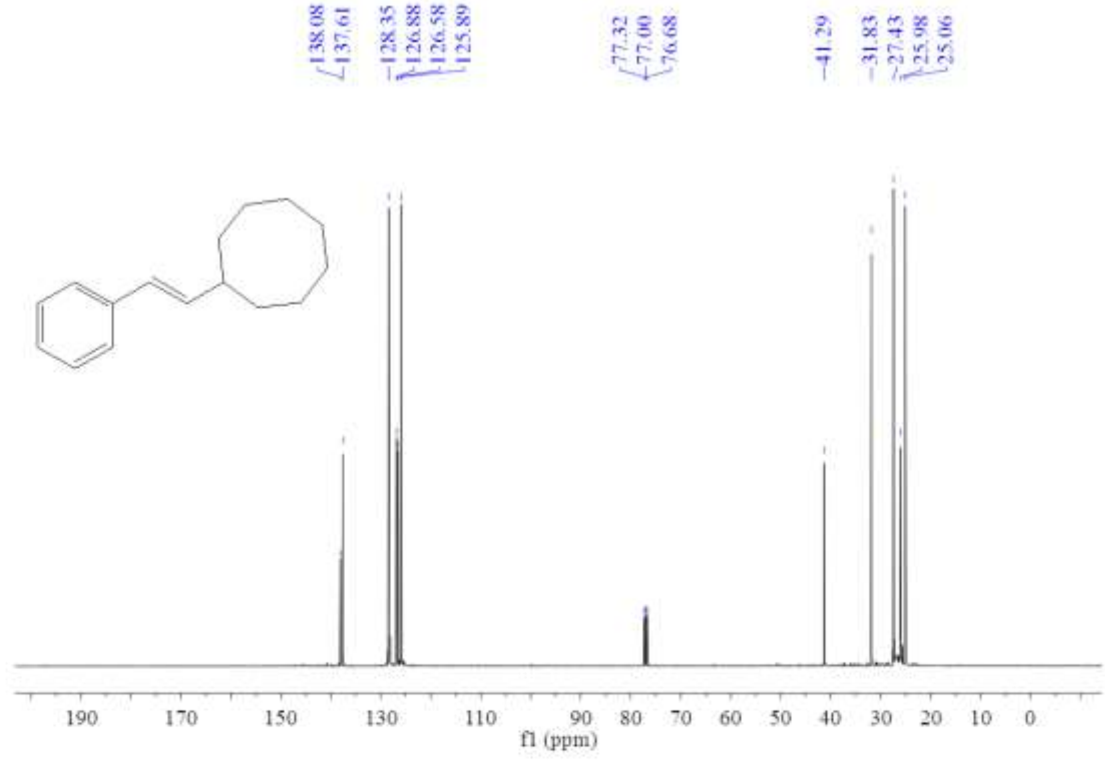

4. References

1. Jang, Y.-J.; Shih, Y.-K.; Liu, J.-Y.; Kuo, W.-Y.; Yao, C.-F. *Chem. Eur. J.* **2003**, *9*, 2123-2128.

2. Ikeda, Y.; Nakamura, T.; Yorimitsu, H.; Oshima, K., *J. Am. Chem. Soc.* **2002**, *124*, 6514-6515.

3. Yao, Q.; Kinney, E. P.; Yang, Z., *J. Org. Chem.* **2003**, *68*, 7528-7531.

4. Loeffler, L. J. and Delorefice, S. B., J. Pharm. Sci. **1975**, *64*, 1170-1173.

5. Ishikawa, T.; Nonaka, S.; Hirao, T., *Chem. Commun.* **1998**, 1209-1210.
